# Prediction of malignancy risk in Bethesda III nodules: development and validation of multiple machine learning models

**DOI:** 10.3389/fendo.2025.1655828

**Published:** 2025-08-29

**Authors:** Wentian Li, Jiayu Zhu, Ying Wang, Jingxiu Li, Zhonghui Li, Cuicui Wang, Jingli Xue, Peng Zhou, Qingqing He

**Affiliations:** ^1^ Shandong First Medical University (Shandong Academy of Medical Sciences), Jinan, Shandong, China; ^2^ Department of Thyroid & Breast Surgery, The 960th Hospital of the PLA Joint Logistics Support Force, Jinan, China; ^3^ Shandong University of Traditional Chinese Medicine, Jinan, Shandong, China; ^4^ Department of Pathology, The 960th Hospital of the PLA Joint Logistics Support Force, Jinan, China

**Keywords:** thyroid nodules, risk of malignancy, atypia of undetermined significance, machine learning, prediction model

## Abstract

**Objective:**

To develop and validate a machine learning (ML)-based prediction model of Bethesda III nodules and create a nomogram based on the best model.

**Methods:**

We collected data on patients with Bethesda III nodules who were admitted between January 2020 and June 2024, including 276 Bethesda III nodules from 7371 patients who underwent ultrasound-guided fine needle aspiration (US-FNA). Clinical, ultrasonographic, cytological, laboratory, and molecular data were collected and randomly split into training and validation cohorts at a ratio of 7: 3. Six feature selection methods and ML algorithms—Logistic Regression (LR), Decision Tree (DT), Random Forest (RF), Extreme Gradient Boosting (XGB), Support Vector Machine (SVM), and K-Nearest Neighbors (KNN)—were evaluated. A nomogram was then created based on the best-performing model.

**Results:**

The study cohort included 276 Bethesda III nodules with a final malignancy rate of 65.2% (180/276). LR exhibited the highest area under the receiver operating characteristic (ROC) curve (AUC: 0.823) in cross-validation of the validation set. Additionally, the calibration curves and Decision Curve Analysis (DCA) results were also favorable. The model included BRAF, composition, shape, orientation, and the thyroid imaging reporting and data system (TI-RADS). The nomogram exhibited robust discrimination (AUC: 0.846 in the validation set), calibration, and clinical applicability across the two datasets after 500 bootstraps.

**Conclusion:**

Among the six ML algorithms, the LR algorithm demonstrated the best performance. A nomogram was developed to predict the malignancy risk in Bethesda III nodules. This nomogram may serve as a valuable tool to reduce diagnostic uncertainty and provide personalized risk stratification for patients.

## 1 Introduction

Thyroid nodules affect approximately 25% of the general population, making them a prevalent clinical condition. The widespread use of high-resolution ultrasound has significantly increased the detection rate of thyroid nodules, with 65% of individuals identified with nodules during ultrasound examinations (1, 2). Although most of these nodules are benign, it remains critical to accurately distinguish malignant lesions to avoid overtreatment and ensure timely intervention. The Bethesda System for Reporting Thyroid Cytopathology (TBSRTC) categorizes fine needle aspiration results into six classifications: (I) non-diagnostic; (II) benign; (III) atypia of undetermined significance (AUS); (IV) follicular neoplasm; (V) suspicious for malignancy; and (VI) malignant. Bethesda III nodules present consistent diagnostic challenges among these categories (3). The frequency of Bethesda III nodules diagnoses varies significantly across laboratories, ranging from 8.6% to 25.3%. While the Bethesda system estimates the malignancy risk for Bethesda III nodules to range from 13% to 30%, clinical data suggest that the actual malignancy rate may be higher, between 23.5% and 40% (4–7). The complexity and uncertainty of Bethesda III nodules underscore the urgent need for comprehensive studies to identify preoperative predictors for these nodules.

Preoperative prediction of the benign or malignant nature of Bethesda III nodules necessitates a comprehensive evaluation that incorporates ultrasound features, molecular testing, thyroid function assessments, and demographic factors such as age and gender. While these factors provide some predictive value for the malignancy of Bethesda III nodules, they each have inherent limitations. For example, the BRAF gene is a highly specific biomarker for papillary thyroid carcinoma (PTC), with a malignancy risk of 98.9% to 100%. However, its mutation rate is only 35% to 77% (8–10), and malignancy is particularly difficult to exclude in follicular subtype Bethesda III nodules when assessed in isolation (11). Molecular testing using a 12-gene panel can improve the diagnostic accuracy of Bethesda III nodules by detecting key mutations such as BRAF, TERT promoter, NRAS/HRAS/KRAS, PIK3CA, AKT1, RET fusions, NTRK fusions, and PAX8-PPARγ. Bethesda III nodules with negative genetic mutations from this panel exhibit a 6% malignancy risk, with less than a 1% risk of cancer spreading beyond the thyroid (12, 13). However, the high cost of the 12-gene panel limits its broader application in China. Thus, a combined approach integrating demographic, imaging, and molecular data may enhance diagnostic accuracy. To our knowledge, there are still few studies specifically addressing the prediction of malignancy risk in Bethesda III nodules (14–16).

Machine learning is the scientific study of algorithms and statistical models that enable computers to perform specific tasks without explicit programming, offering notable advantages in the field of thyroid surgery (17). Emerging evidence suggests multimodal GPTs may improve diagnostic accuracy through integrated data analysis (18–20). Radiomics has recently demonstrated effectiveness in automating thyroid nodule classification and risk stratification (16). Compared to traditional logistic regression models, ML excels in handling complex clinical data, facilitating the development of predictive tools that, in specific cases, outperform conventional statistical models (21). Although AI research in thyroidology continues to advance, there is limited exploration focused on the most clinically challenging Bethesda III nodules. Recent studies have nevertheless achieved promising results in predicting malignancy for these nodules using machine learning. Cao et al. (14) developed a nomogram with 0.80 AUC by comparing machine learning methods to logistic regression, whereas Zhong et al. (16) reported 0.823 AUC but with narrower applicability. Our study employs machine learning algorithms to integrate multidimensional data, including ultrasound features, clinical parameters, and molecular biomarkers, to develop a personalized malignancy risk prediction model for Bethesda III nodules. Through systematic comparison of advanced algorithms with LR, we aim to develop a visual decision support tool that reduces diagnostic uncertainty, prevents unnecessary surgeries, and provides personalized risk stratification for patients.

## 2 Materials and methods

### 2.1 Study cohort

This retrospective observational study was approved by the 960th Hospital of the PLA Joint Logistics Support Force Research Ethics Committee (No. 2024-171). Research participants were performed in accordance with the Declaration of Helsinki, and each patient provided written informed consent. All eligible patients were informed about the use of their data for study and had the option to decline to participate (22).

This study included patients with thyroid nodules diagnosed as Bethesda III nodules based on initial FNA between January 2020 and June 2024. Final diagnoses were confirmed through repeat biopsies, 12-gene testing, or surgery (23). Inclusion criteria included: (1) thyroid nodules classified as Bethesda III on FNA cytology; (2) preoperative ultrasound, molecular testing, and thyroid function tests; (3) patients underwent thyroidectomy; (4) at least one repeat FNA on the same Bethesda III nodule within one year; (5) 12-gene panel testing. Exclusion criteria included: (1) other thyroid cancer types, such as follicular carcinoma or medullary thyroid carcinoma; (2) patients lacking laboratory, imaging, or pathological data; (3) uncertain repeat FNA results; (4) any mutations detected in the 12-gene test; (5) patients without at least six months of follow-up after repeat FNA or 12-gene testing. Patients were randomly assigned to a training set and a validation set in a 7:3 ratio. The training set was used to develop models with various machine learning algorithms, and the validation set was used to evaluate model performance.

### 2.2 Clinical features and data collection

Data for the variables assessed in this study were collected from patients’ hospitalization electronic medical records (EMRs), including basic patient information, thyroid imaging reporting and data system, cytological assessments, molecular testing following US-FNA, laboratory indicators (within one month before surgery) and postoperative pathological data. Basic patient information included age and sex. Ultrasound features of thyroid nodules were analyzed using TI-RADS terminology, including size, composition, echogenicity, margin, shape, echogenic foci, halo, orientation, location, color Doppler flow imaging (CDFI) pattern, echotexture, posterior features, cervical lymph nodes and solitary nodule (24). The largest transverse, anteroposterior, and vertical diameters of all nodules were recorded, with the largest of these three measurements used to assess nodule size. Additional features of the nodule were recorded according to C-TIRADS. C-TIRADS classified the nodules by assigning points for composition, echogenicity, shape, margin, and echogenic foci to determine the TIRADS level. Cervical lymph nodes were considered abnormal if they exhibited any of the following features: (a) loss of central hilar echo, (b) cystic change, (c) calcification, (d) cortical hyperechogenicity, (e) increased and irregular vascularity, or (f) cervical lymphadenopathy. Two radiologists reviewed and documented all sonographic characteristics of the thyroid nodules. Disagreements were resolved through discussion or consultation with a third radiologist. In cytological assessments and molecular testing, cytologists examined the cells and cellular structures according to the second edition of the Bethesda System for Reporting Thyroid Cytopathology (3). Bethesda III cases were reviewed and categorized into the following subtypes: (1) AUS-nuclear atypia: Includes focal nuclear atypia or mild but extensive nuclear atypia; (2) AUS-other: Includes architectural atypia (often sparsely cellular samples predominantly composed of microfollicles) and Hurthle cell atypia (oncocytic features). Two molecular tests were available at our institution: BRAF and a twelve-gene molecular panel. Patients underwent twelve-gene panel testing, which included BRAF, RAS (NRAS, HRAS, KRAS), PIK3CA, AKT1, RET, CCDC6-RET, NCOA4-RET, TP53, ETV6-NTRK3, TPM3-NTRK1, TERT, and PAX8-PPARG promoters. Laboratory indicators obtained included thyroid stimulating hormone (TSH), free thyroxine (FT4), free triiodothyronine (FT3), thyroglobulin antibody (TgAb), thyroid peroxidase antibody (TPOAb), and thyroglobulin (Tg) levels. All predictive factors were derived from objective data and image archiving in the EMRs.

### 2.3 Assessment of study outcomes

The definitive diagnosis of malignant tumors was confirmed through histopathological analysis of surgically excised tissue or repeated fine-needle aspiration biopsies with malignant results (Bethesda categories V or VI). In contrast, benign nodules were diagnosed based on one of the following criteria: (1) histopathological confirmation from surgically excised specimens, (2) absence of mutations in a 12-gene panel, or (3) at least one benign FNA result (Bethesda II) from repeated biopsies, with no subsequent malignant findings. All patients who did not undergo surgical intervention were followed up with ultrasonography for at least six months to assess any suspicious changes in the nodules. Histopathological results were independently reviewed by two experienced pathologists, and any discrepancies were resolved through consultation with a third pathologist.

### 2.4 Sample size calculation

We calculated the required sample size using the events per variable (EPV) metric, a widely recognized method in statistical analysis (25–27). We also followed the 4-step procedure proposed by Riley et al. (28) to calculate the required sample size (29). According to guidelines, the malignancy rate of Bethesda III nodules typically ranges from 13% to 30% based on follow-up of surgically resected nodules (3). Since we intended to include patients who had not undergone surgery, the expected proportion of the endpoint event was estimated at 0.15. The calculation process, formulas, and results are shown in **Supplementary Table S2**.

### 2.5 Data preprocessing

Statistical analyses were conducted using SPSS 25.0 software and R (version 4.4.2; R Foundation, Vienna, Austria), with a significance level set at p < 0.05. The “mice” package in R was used to assess the missing data mechanism, and the VIM package was used for data visualization. Multiple imputation was used to handle missing data, with 10 imputations performed based on the established MAR mechanism, as recommended by standard guidelines. The Multiple Imputation by Chained Equations (MICE) method was used for imputation, implemented through the “mice” package in R. The imputation model with the lowest Bayesian Information Criterion (BIC) was chosen for data optimization. Continuous variables were imputed using predictive mean matching, categorical variables using logistic regression, and multinomial variables using multinomial logistic regression. All relevant covariates, including predictors, outcome variables, and other variables not included in the predictive model, were incorporated into the imputation model to capture the relationships among the variables.

### 2.6 Selection of variables

**Supplementary Figure S1** presents the complete study flowchart. Before constructing the predictive model, six feature selection methods were applied to mitigate the high correlation between predictor variables and improve both the predictive accuracy and interpretability of the model. These methods were chosen based on their proven effectiveness in handling high-dimensional data and identifying the most relevant predictor variables in predictive modeling. In clinical prediction models, the combined use of multiple variable selection methods can compensate for the limitations of individual approaches, thereby enhancing the stability of variable selection and the predictive performance of the model (30, 31). The feature selection methods used include:

1. Stepwise Regression (SR): This method uses a stepwise selection mechanism to dynamically adjust explanatory variables in the model based on hypothesis testing results, effectively extracting core influencing factors while alleviating collinearity issues. We implemented three variations of stepwise regression: forward selection (FS), backward selection (BS), and bidirectional elimination (BE).
2. Least Absolute Shrinkage and Selection Operator (LASSO): This method performs variable selection and regularization by adding an L1 penalty to the regression coefficients. It is particularly effective for high-dimensional data, especially when dealing with small sample sizes and high multicollinearity between predictor variables (32).
3. Boruta: Built upon the random forest framework, this algorithm constructs a feature evaluation mechanism by comparing the dynamic importance between original variables and shadow variables, using two-tailed statistical validation to achieve precise separation of feature signals and ensuring the retention of all relevant features.
4. Random Forest-Recursive Feature Elimination (RF-RFE): This method uses an iterative elimination strategy to optimize feature subsets, ensuring their relevance to the classifier and thereby improving model performance.

We summarized 29 candidate predictors selected through various filtering methods and took their intersection. Finally, we consulted with clinical experts and combined them with clinical reality to determine the final predictors for constructing Bethesda III nodules malignancy prediction model.

### 2.7 Model development and performance comparison

Six machine learning models were used to predict the malignancy of Bethesda III nodules: LR (glm stats package), DT (rpart package), RF (randomForest package), XGB (xgboost package), SVM (e1071 package), and KNN (kknn package). The selected clinical features were fed into the six models using various machine learning algorithms. To evaluate the models’ performance on unseen data, five-fold cross-validation was applied to obtain the parameters. Performance metrics from the confusion matrix were used to assess model efficacy in both the training and validation cohorts, including the Receiver Operating Characteristic curve, sensitivity (SEN), specificity (SPE), positive predictive value (PPV), negative predictive value (NPV), accuracy (ACC), F1 score, and Brier score. A calibration curve was used to compare predicted probabilities with actual outcomes. Decision Curve Analysis was used to evaluate the net benefit of the models at various thresholds. Additionally, the DeLong test was used to determine whether significant differences existed in the AUC values of the models. Based on the evaluation of these metrics in both the training and testing sets, the optimal model was selected.

### 2.8 Model explanation

LR was selected as the optimal model based on its performance. The model’s performance was demonstrated using 500 bootstrap samples, including the ROC curve, calibration curve, and DCA results. To assess model interpretability and evaluation, we calculated the AUC to evaluate the model’s discriminative ability and examined its calibration using the Hosmer-Lemeshow (HL) test. DCA was used to evaluate the net benefit at different thresholds, providing a comprehensive assessment of the model’s effectiveness in real-world medical decision-making scenarios. Bootstrapping, a statistical method, was applied to estimate model accuracy by repeatedly sampling with replacement from the original dataset. The model was trained on these new datasets and evaluated using out-of-bag data. This process was repeated several times to obtain a distribution of performance metrics, yielding a robust estimate of model reliability and variance. The nomogram score is the sum of the scores assigned to each risk factor, where higher scores indicate a greater risk of Bethesda III nodules malignancy. This graphical tool simplifies the estimation of individual risk or probability based on the variables predicted by the model.

### 2.9 Statistical analysis

All analyses were conducted in R. For continuous variables, the Shapiro-Wilk test was used to assess their normality. This test was selected because it generally performs better than other tests, such as the Kolmogorov-Smirnov test, particularly with small sample sizes. Variables following a normal distribution were described using the mean ± standard deviation, while non-normally distributed variables were described using the median and interquartile range, providing more robust measures of central tendency and variability in the presence of outliers. Frequency and percentage were reported for categorical variables. To assess collinearity between variables, the variance inflation factor (VIF) was calculated. A VIF value below 5 and tolerance greater than 0.1 typically indicate no significant collinearity between variables. This metric helps ensure that our regression models are not unduly affected by multicollinearity, which can distort the estimated relationships between predictors and outcomes. Fisher’s exact test was used for categorical variables with low expected frequencies to ensure accurate significance testing. This test provides a precise method for determining the likelihood of observing a given set of frequencies in categorical variables, particularly useful with small sample sizes. Several R packages were used for specific analyses: comparegroups for baseline description, glmnet for LASSO regression, forestmodel for forest plots, pROC, ggROC, and fbroc for discriminative analysis, PRROC for PR curves, rms for calibration using val.prob and calibrate functions, ResourceSelection for the Hosmer-Lemeshow test, dcurves for DCA, and rms for nomogram construction.

## 3 Result

### 3.1 Study cohort and baseline information

Between January 2020 and June 2024, 7,371 patients underwent ultrasound-guided fine needle aspiration, while 2,761 patients underwent thyroidectomy. Among the thyroid nodules assessed by US-FNA, 10.2% (754/7,398) were diagnosed with Bethesda III nodules. Of these Bethesda III nodules, 31.6% (238/754) underwent surgical resection, 7.3% (55/754) underwent repeat biopsies, and 6.9% (52/754) were subjected to 12-gene panel testing. Notably, 36.4% (20/55) of the Bethesda III nodules that underwent repeat biopsies had indeterminate results. A total of 69 patients were excluded due to incomplete records (16), other pathologically confirmed thyroid cancer types (2), uncertain repeat biopsy outcomes (20), mutations found by 12-gene panel testing (1), or insufficient follow-up for at least six months after repeat biopsy or 12-gene panel testing (30). Consequently, the current study included 271 patients: 213 females and 58 males, with a total of 276 Bethesda III nodules. Approximately 65.2% (180/276) of the nodules were malignant, while 34.8% (96/276) were benign. BRAF mutations were detected in 41% (113/276) of the cases. In our dataset, only 36 cases (13.04%) were classified as AUS and 25 (9.06%) as FLUS. Neither AUS nor FLUS showed statistical significance in the univariate analysis. The median age of patients was 51 years (IQR 41.00–57.25), and the median nodule size was 5.2 mm (IQR 4.00–7.70). In the current dataset, five variables were missing, all related to thyroid function tests, resulting in 232 missing values, or approximately 2.8% of the total data points. These missing values were distributed among multiple variables, as detailed in **Supplementary Table S1** and **Supplementary Figure S2**. The cohort was randomly divided into a training set (n = 193) and a validation set (n = 83). As shown in **Table 1**, no significant differences were observed in the baseline characteristics between the two groups (P > 0.05).

**Table 1:** Baseline characteristics of all patients between the training and validation cohorts.

Characteristics	Total N=276	Training N=193	Testing N=83	P value
Gender (%)				0.271
Male	60 (21.74)	38 (19.69)	22 (26.51)	
Female	216 (78.26)	155 (80.31)	61 (73.49)	
Age (median [IQR])	51.00[41.00;57.25]	51.00[41.00;58.00]	50.00 [36.00;56.50]	0.245
Nuclear atypia (%)				0.195
Present	36 (13.04)	29 (15.03)	7 (8.43)	
Absent	240 (86.96)	164 (84.97)	76 (91.57)	
Architectural atypia (%)				0.356
Present	25 (9.06)	20 (10.36)	5 (6.02)	
Absent	251 (90.94)	173 (89.64)	78 (93.98)	
BRAF^V600E^ (%)				1.000
Positive	113 (40.94)	79 (40.93)	34 (40.96)	
Negative	163 (59.06)	114 (59.07)	49 (59.04)	
Diameter (median [IQR])	5.20 [4.00;7.70]	5.30 [4.00;8.10]	5.00 [4.00;7.00]	0.513
Solid composition (%)				0.188
Yes	236 (85.51)	161 (83.42)	75 (90.36)	
No	40 (14.49)	32 (16.58)	8 (9.64)	
Nodule position 1(%)				0.227
Left lobe	134 (48.55)	98 (50.78)	36(43.37)	
Right lobe	133 (48.19)	88 (45.60)	45 (54.22)	
Isthmus	9 (3.26)	7 (3.63)	2 (2.41)	
Marked Hypoechoic(%)				0.687
Yes	21 (7.61)	16 (8.29)	5 (6.02)	
No	255 (92.39)	177 (91.71)	78 (93.98)	
Unclear boundary (%)				1.000
Yes	227 (82.25)	159 (82.38)	68 (81.93)	
No	49 (17.75)	34 (17.62)	15 (18.07)	
Irregular shape (%)				0.532
Yes	58 (21.01)	43 (22.28)	15 (18.07)	
No	218 (78.99)	150 (77.72)	68 (81.93)	
Microcalcification (%)				1.000
Yes	44 (15.94)	31 (16.06)	13 (15.66)	
No	232 (84.06)	162 (83.94)	70 (84.34)	
Calcification (%)				0.643
No calcification	159 (57.61)	112 (58.03)	47 (56.63)	
Microcalcification	69 (25.00)	50 (25.91)	19 (22.89)	
Macrocalcification	48 (17.39)	31 (16.06)	17 (20.48)	
Halo (%)				0.384
Present	32 (11.59)	25 (12.95)	7 (8.43)	
Absent	244 (88.41)	168 (87.05)	76 (91.57)	
Orientation (%)				0.853
Taller-than-wide	129 (46.74)	89 (46.11)	40 (48.19)	
Wider-than-tall	147 (53.26)	104 (53.89)	43 (51.81)	
Upper region (%)				0.733
Yes	58 (21.01)	39 (20.21)	19 (22.89)	
No	218 (78.99)	154 (79.79)	64 (77.11)	
Nodule Position 2(%)				0.835
Upper region	58 (21.01)	39 (20.21)	19 (22.89)	
Middle region	135 (48.91)	93 (48.19)	42 (50.60)	
Lower region	73 (26.45)	53 (27.46)	20 (24.10)	
Isthmus	10 (3.62)	8 (4.15)	2 (2.41)	
CDFI pattern (%)				0.988
Present	65 (23.55)	46 (23.83)	19 (22.89)	
Absent	211 (76.45)	147 (76.17)	64 (77.11)	
Echotexture (%)				0.168
Homogeneous	98 (35.51)	63 (32.64)	35 (42.17)	
Heterogeneous	178 (64.49)	130 (67.36)	48 (57.83)	
Posterior features (%)				0.546
Present	65 (23.55)	43 (22.28)	22 (26.51)	
Absent	211 (76.45)	150 (77.72)	61 (73.49)	
Shadowing Posterior features (%)				1.000
Yes	75 (27.17)	52 (26.94)	23 (27.71)	
No	201 (72.83)	141 (73.06)	60 (72.29)	
Suspicious LN (%)				0.269
Yes	100 (36.23)	68 (35.23)	32 (38.55)	
No	176 (63.77)	125 (64.77)	50 (61.45)	
TI-RADS (%)				0.425
3	53(19.20)	39 (20.21)	14(16.87)	
4a	102(36.96)	64(33.16)	38(45.78)	
4b	78(28.26)	57(29.53)	21(25.30)	
4c	29(10.51)	22(11.40)	7(8.43)	
5	14(5.07)	11 (5.70)	3 (3.61)	
Solitary nodule (%)				0.395
Yes	112 (40.58)	82 (42.49)	30 (36.14)	
No	164 (59.42)	111 (57.51)	53 (63.86)	
TSH (median [IQR])	1.76 [1.08;2.58]	1.77 [1.03;2.58]	1.74 [1.11;2.55]	0.745
FT4(median [IQR])	13.58 [11.63;16.24]	13.68 [11.70;16.60]	13.40 [11.59;15.64]	0.199
FT3(median [IQR])	4.49 [4.07;5.01]	4.48 [4.10;5.01]	4.50 [4.02;5.00]	0.882
TgAb (median [IQR])	7.61 [1.15;19.88]	10.80 [1.22;20.50]	3.28 [1.00;18.45]	0.130
TPOAb (median [IQR])	7.08 [1.00;14.98]	9.00 [1.04;15.50]	2.13 [1.00;12.70]	0.173

CDFI, Color Doppler Flow Imaging; LN, Lymph Node; TI-RADS, Thyroid Imaging Reporting and Data System; TSH, Thyroid-Stimulating Hormone; FT4, Free Thyroxine; FT3, Free Triiodothyronine; TgAb, Thyroglobulin Antibody; TPOAb, Thyroid Peroxidase Antibody.

### 3.2 Selection of clinical characteristics

**Figure 1:**
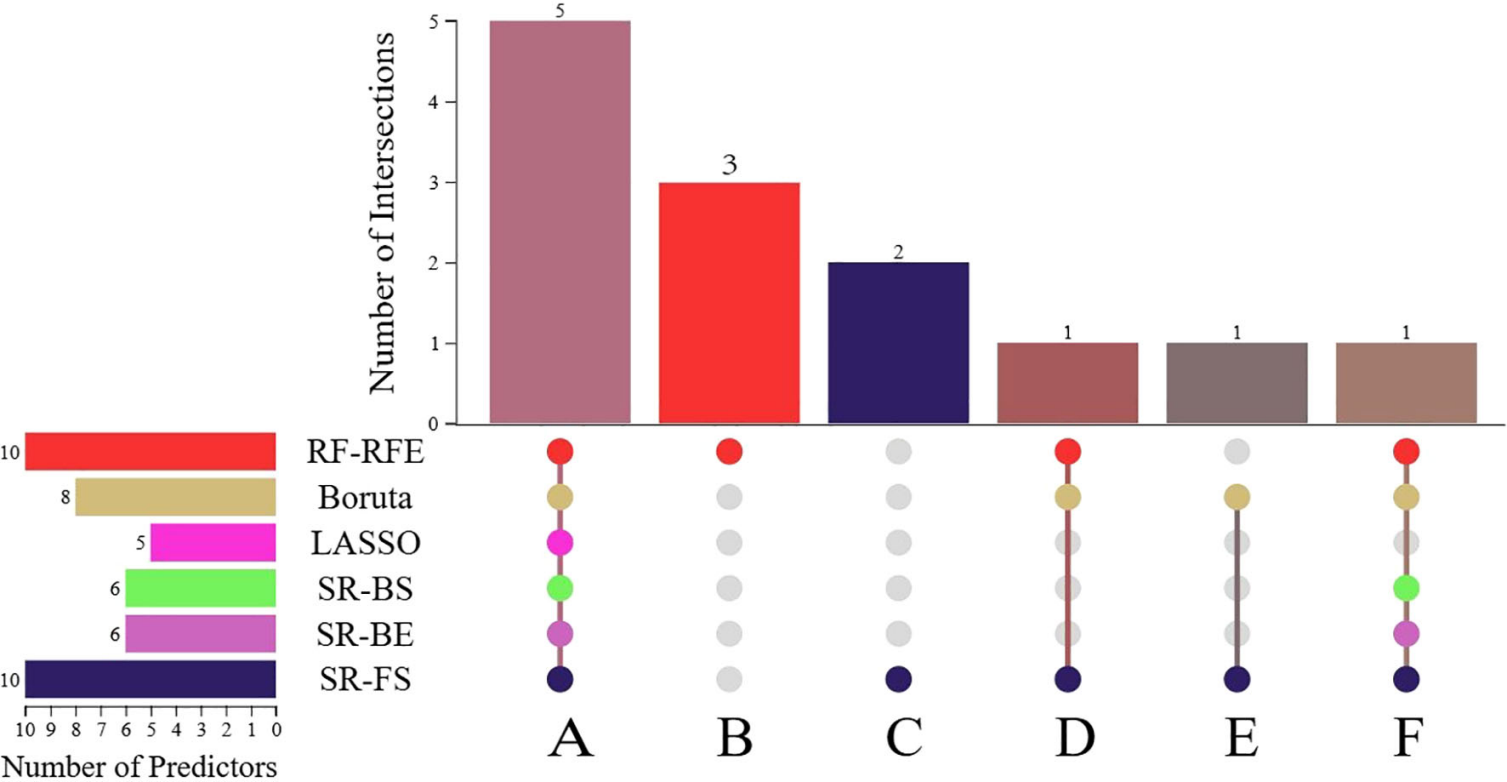
Upset plot of interactions between the predictors. **(A)** BRAF, composition, shape, orientation, TI-RADS. **(B)** TSH, FT4, Echotexture. **(C)** AUS, Echogenicity. **(D)** Size. **(E)** Margin. **(F)** TgAb. SR-FS, stepwise regression-forward selection; SR-BS, stepwise regression-backward selection; SR-BE, stepwise regression-bidirectional elimination; LASSO, least absolute shrinkage and selection operator; RF-RFE, random forest -recursive feature elimination.

The predictor variables selected by the six methods are presented in **Table 2**. Detailed parameters for all methods (SR-FS, SR-BS, SR-BE, LASSO, Boruta and RF-RFE) can be found in **Supplementary Table S4** and **Supplementary Figures S3**-**S5**. A total of 10, 6, 6, 5, 8, and 10 predictors were identified using the six selection methods. As shown in **Supplementary Table S4**, variables such as TSH, FT4, and Echotexture did not show a significant association with malignancy in the initial univariate screening and were therefore not carried forward into the final model building stage. **Figure 1** illustrates the intersection of predictors selected by the six methods. Predictors with more than six intersections were selected as final predictors, including BRAF, composition, shape, orientation, and TI-RADS. Ultimately, five predictors were included in the development of the model.

**Table 2:** Predictor variables of Bethesda III nodules using different selection methods.

Methods	Predictor variables
SR-FS	AUS, BRAF, Size, Composition, Echogenicity, Margin, Shape, Orientation, TI-RADS, TgAb
SR-BE	BRAF, Composition, Shape, Orientation, TI-RADS, TgAb
SR-BS	BRAF, Composition, Shape, Orientation, TI-RADS, TgAb
LASSO	BRAF, Composition, Shape, Orientation, TI-RADS
Boruta	BRAF, Size, Composition, Margin, Shape, Orientation, TI-RADS, TgAb
RF-RFE	BRAF, Size, Composition, Shape, Orientation, TI-RADS, TgAb, TSH, FT4, Echotexture

SR-FS, stepwise regression-forward selection; SR-BS, stepwise regression-backward selection; SR-BE, stepwise regression-bidirectional elimination; LASSO, least absolute shrinkage and selection operator; RF-RFE, random forest feature elimination; AUS, Atypia of Undetermined Significance; TI-RADS, Thyroid Imaging Reporting and Data System; TSH, Thyroid-Stimulating Hormone; FT4, Free Thyroxine; TgAb, Thyroglobulin Antibody.

### 3.3 Performances of different models

**Figure 2:**
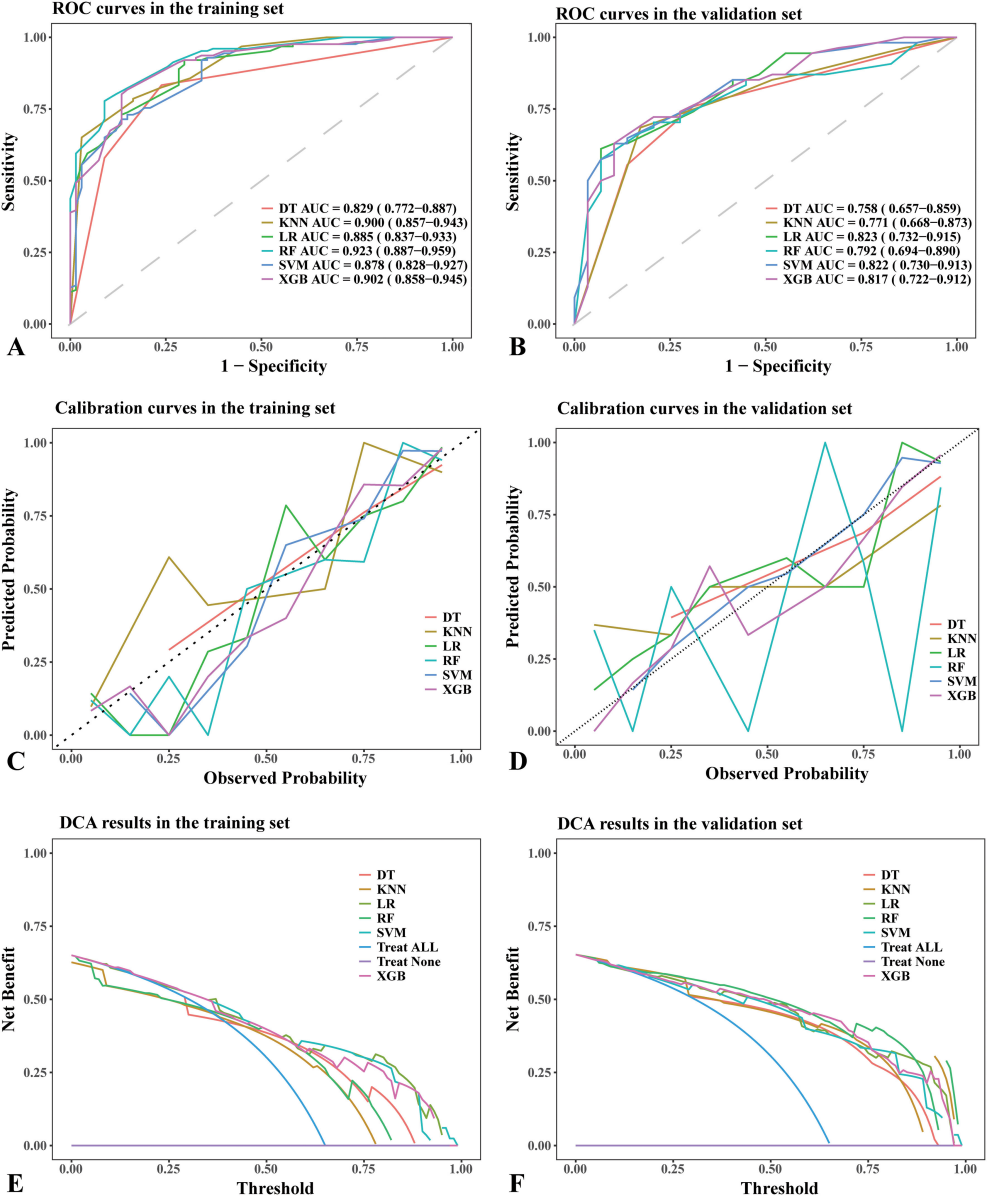
Performance comparison of different machine learning models in the training set and validation set. **(A)** ROC curves in the training set. **(B)** ROC curves in the validation set. **(C)** Calibration curves in the training set. **(D)** Calibration curves in the validation set. **(E)** DCA results in the training set. **(F)** DCA results in the validation set. **(A, B)** The dotted lines in the ROC curves represent the reference line. **(C, D)** The dotted lines in the calibration curves represent a perfect prediction by an ideal model. **(E, F)** The “treat all” lines in the DCA results assume that all nodules were malignant, whereas the “treat none” lines assume that all nodules were benign lesions. Abbreviations: DT, decision tree; AUC, area under curve; KNN, k-nearest neighbors; LR, logistic regression; RF, random forest; SVM, support vector machine; XGB, XGBoost; ROC, receiver operating characteristic; DCA, Decision Curve Analysis.

In our comparative analysis, we evaluated five machine learning models against the LR model. Although complex models such as RF achieved better training set performance, the LR model exhibited the most robust and clinically meaningful results in the validation set, as evidenced by **Table 3** and **Figure 2**. The six models were developed using six distinct machine learning algorithms. The estimated odds ratios for the logistic regression model are presented in **Supplementary Table S5** and visualized in a forest plot (**Supplementary Figure S6**). We further illustrate visualizations for the other models, including the relative importance of potential features and heatmaps of confusion matrices for the decision tree, random forest, extreme gradient boosting, support vector machine, and k-nearest neighbors models derived from the training cohort. However, due to the nature of the KNN model, the ranking of feature importance is not applicable in this case.

**Table 3:** Predictive performance metrics of different machine learning algorithms of the training set and validation set.

Model	LR	DT	RF	XGB	SVM	KNN
Training set						
AUC (95%)	0.89 (0.84-0.93)	0.83 (0.77-0.89)	0.92 (0.89-0.96)	0.90 (0.86-0.95)	0.88 (0.83-0.93)	0.90 (0.86-0.94)
SEN	0.92	0.87	0.78	0.82	0.73	0.79
SPE	0.70	0.71	0.91	0.85	0.85	0.84
PPV	0.85	0.83	0.94	0.91	0.90	0.90
NPV	0.82	0.76	0.69	0.71	0.63	0.68
ACC	0.85	0.83	0.94	0.91	0.90	0.90
F1 score	0.89	0.85	0.85	0.86	0.81	0.84
Brier score	0.13	0.15	0.11	0.12	0.13	0.14
Validation set						
AUC (95%)	0.82 (0.73-0.92)	0.76 (0.66-0.86)	0.79 (0.69-0.89)	0.82 (0.72-0.91)	0.82 (0.73-0.91)	0.77 (0.67-0.87)
SEN	0.85	0.82	0.70	0.74	0.63	0.80
SPE	0.55	0.61	0.72	0.72	0.86	0.59
PPV	0.78	0.76	0.83	0.83	0.90	0.78
NPV	0.67	0.69	0.57	0.60	0.56	0.61
ACC	0.78	0.76	0.83	0.83	0.90	0.78
F1 score	0.81	0.79	0.76	0.78	0.74	0.79
Brier score	0.16	0.19	0.21	0.16	0.16	0.23

DT, decision tree; KNN, k-nearest neighbors; LR, logistic regression; RF, random forest; SVM, support vector machine; XGB, XGBoost; AUC, area under curve; SEN, sensitivity; SPE, specificity; PPV, positive predictive value; NPV, negative predictive value; ACC, accuracy.

The RF model demonstrated the highest area under the ROC curve (AUC, 0.923; 95% CI: 0.887–0.959) in the training set (**Figure 2A**), whereas the LR model achieved the highest AUC (0.823; 95% CI: 0.732–0.915) in the validation set (**Figure 2B**). The AUC values for the remaining models in the validation set were as follows: (1) DT: 0.758, 95% CI: 0.657–0.859; (2) RF: 0.792, 95% CI: 0.694–0.890; (3) XGB: 0.817, 95% CI: 0.722–0.912; (4) SVM: 0.822, 95% CI: 0.730–0.913; (5) KNN: 0.771, 95% CI: 0.668–0.873 (**Figure 2B**). Based on the DeLong test (**Supplementary Tables S7**, **S8**), no significant difference in AUC was observed in the validation cohort. This may be due to the limited sample size or the similar performance of the models.

The DT model showed the best consistency between observed and predicted results in both the training and validation sets (**Figure 2C**). In contrast, the LR, SVM, KNN, and XGB models exhibited similarly good consistency between observed and predicted results in the validation set (**Figure 2D**). The consistency between observed and predicted results in the RF model was less stable in the validation set (**Figure 2D**).

In the training set, all models demonstrated similar DCA results (**Figure 2E**), whereas the LR, SVM, and XGB models achieved the best DCA outcomes in the validation set (**Figure 2F**). In the validation set, using alternative models resulted in a greater net benefit compared to no treatment or full treatment strategies when the threshold probability was <80%.

The RF model exhibited the highest precision and specificity in the training set, whereas the LR model achieved the highest recall and F1 score, as well as the second lowest Brier score. In the validation set, the LR model achieved the highest recall and F1 score, whereas the SVM model demonstrated the highest precision. Detailed information can be found in **Table 3**. Model performance in the validation set served as the primary criterion for identifying the optimal model. Although the LR model exhibited moderate specificity (0.55), its superior AUC (0.823), recall (sensitivity, 0.85), and F1 score (0.81) justified its selection as the optimal model. For malignancy risk assessment, minimizing false negatives through high recall remains clinically critical.

In summary, while the RF model may exhibit potential overfitting, the LR model demonstrates not only strong interpretability but also achieves the highest AUC in the validation set. Taking into account model performance, complexity, generalization capability, and practicality, the LR model was ultimately chosen. The forest plot illustrates variables with P-values greater than 0.05 (composition), and due to its clinical significance in daily medical practice, this variable was retained in the logistic regression model for interpretation of its effect size.

### 3.4 Nomogram construction and application

**Figure 3:**
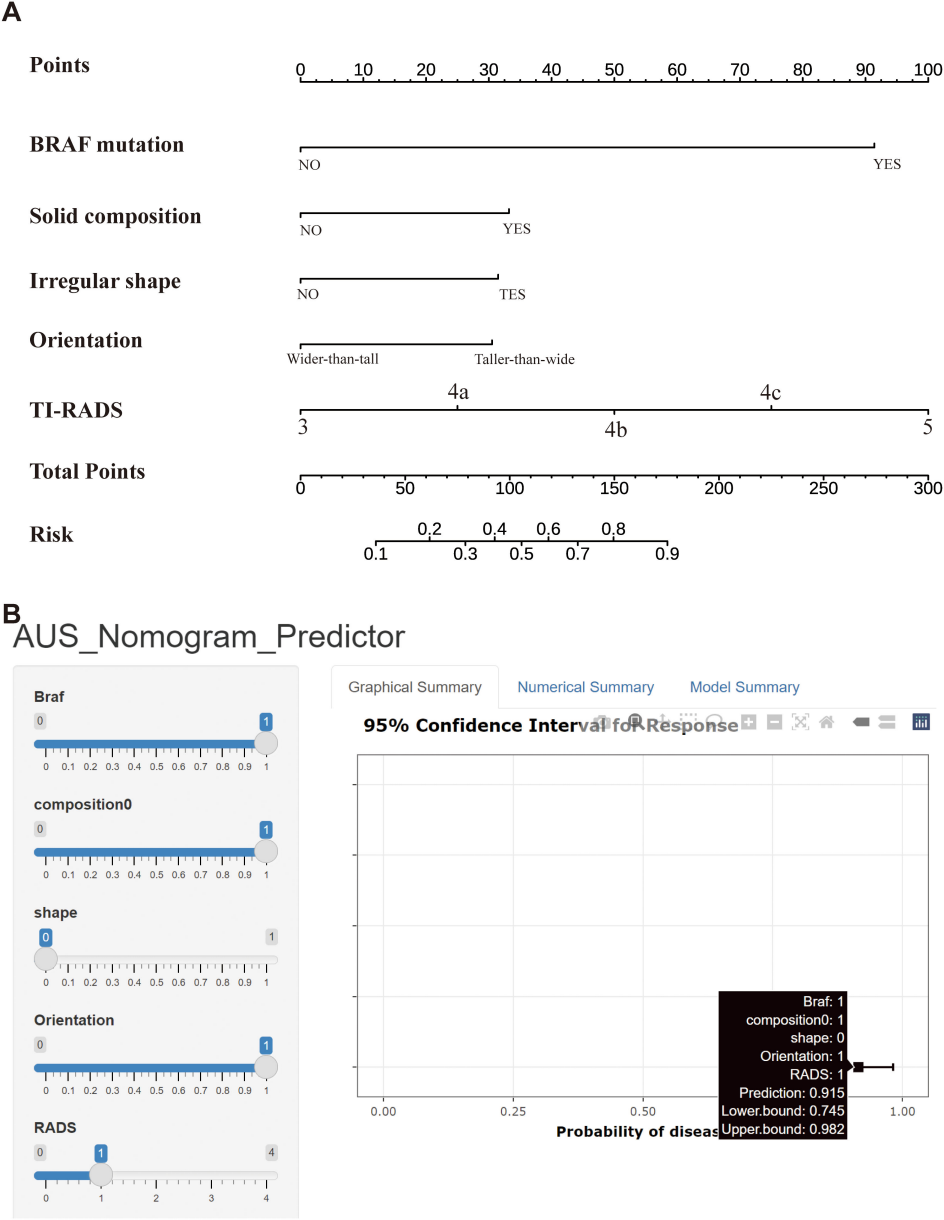
**(A)** Nomogram used for predicting the risk of malignancy for Bethesda III nodules. Logistic regression algorithm was used to establish nomogram. The final score (ie, Total Points) is calculated as the sum of the individual scores of each of the ten variables included in the nomogram. **(B)** A web-based calculator for predicting malignancy risk in Bethesda III nodules. Abbreviations: TI-RADS, Thyroid Imaging Reporting and Data System.

**Figure 4:**
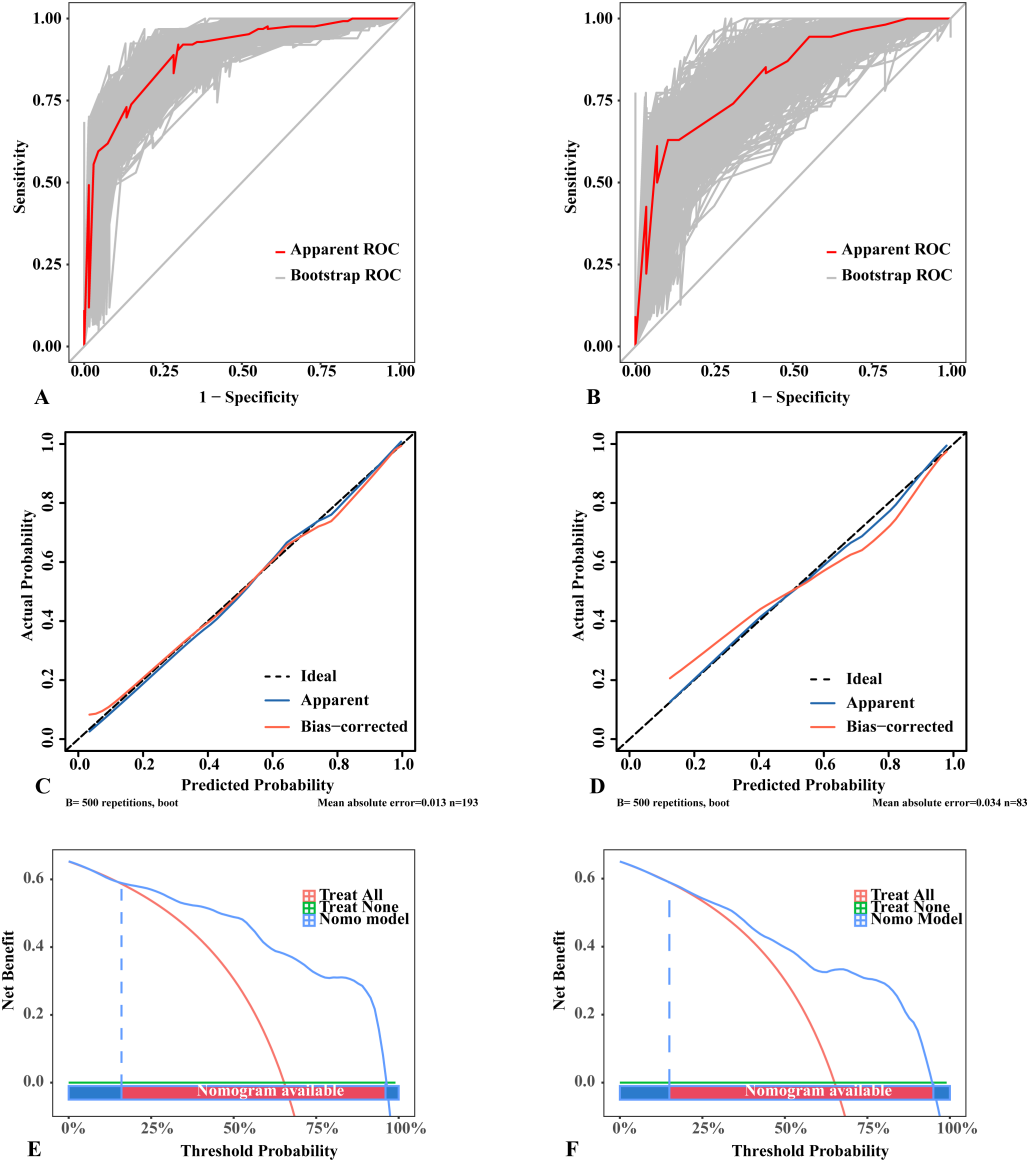
Performance of the logistic regression model with 500 bootstraps. **(A)** ROC curves in the training set. **(B)** ROC curves in the validation set. **(C)** Calibration curves in the training set. **(D)** Calibration curves in the validation set. **(E)** DCA results in the training set. **(F)** DCA results in the validation set. **(A, B)** The diagonal lines in the ROC curves represent the reference line; the “Apparent ROC” lines represent the apparent performance of the model, whereas the “Bootstrap ROC” lines represent the model’s performance after 500 bootstraps. **(C, D)** The dotted lines in the calibration curves represent a perfect prediction by an ideal model; the “Apparent” lines denote the apparent performance of the model, whereas the “Bias-corrected” lines reflect the model’s performance after 500 bootstraps; a closer fit to the dotted lines indicates a better predictive effect. **(E, F)** The “treat all” lines in the DCA results assume that all nodules were malignant, whereas the “treat none” lines assume that all nodules were benign lesions; the “Nomo model” lines represent the performance of the model. For example, if a patient’s threshold probability is 75%, the net benefit is approximately 0.3 in the validation set, meaning that 30 out of 100 patients may benefit from using this model. ROC, receiver operating characteristic; DCA, Decision Curve Analysis.

The nomogram was constructed by incorporating five variables—BRAF, composition, shape, orientation, and TI-RADS—into the predictive model (**Figure 3**). Following 500 bootstrap iterations, the LR model demonstrated an AUC of 0.871 (95% CI: 0.817–0.926) in the training set and 0.846 (95% CI: 0.762–0.930) in the validation set (**Figures 4A, B**). After 500 bootstrap iterations for calibration, the calibration curve closely aligned with the ideal diagonal. The average absolute errors (MAEs) for the two datasets were 0.013 and 0.031, respectively, indicating that the predicted probabilities closely aligned with the actual outcomes across different samples (**Figures 4C, D**). The Hosmer-Lemeshow test confirmed good consistency between the predicted and observed results (P = 0.414). DCA showed that applying the LR nomogram provided greater net benefit than no treatment or full treatment strategies when the threshold probability ranged from 16% to 96% in the training set and from 15% to 95% in the validation set (**Figures 4E, F**). Using this model, clinicians can more accurately assess the risk of malignancy in Bethesda III nodules, providing optimized management and treatment options. The integration of this predictive model significantly enhances the precision of patient management. This highlights the importance of leveraging precise, data-driven decisions in clinical practice. We further assessed the independence of the variables in the model by calculating the VIF and found that all VIF values were well below the common threshold of 5, indicating low multicollinearity and confirming the model’s stability and reliability. The detailed VIF values are provided in **Supplementary Table S6**. Our analysis compared the ROC curves of the nomogram with those of the five individual predictors for malignancy risk assessment. The nomogram exhibited superior diagnostic performance, as illustrated in **Supplementary Figure S14**.

Compared to the complex logistic regression formula, the nomogram is simpler, more intuitive, and clinically practical. To use the nomogram, a line is drawn from the value of each feature to the “points” axis, obtaining the corresponding score. After summing these points, the total score is located on the “total points” axis. Finally, a line is drawn downward from the total score to the “probability of malignancy” axis to determine the corresponding risk. For example, when a patient is BRAF-positive with an ultrasound indicating a solid nodule, a regular shape, a vertical-to-horizontal ratio <1, and a TI-RADS score of 4a, their “BRAF” score is 91, “composition” is 32, “shape” is 0, “orientation” is 0, and “TI-RADS” is 24. The total score is 147, corresponding to a malignancy probability of 0.72 (72%). Therefore, the surgeon may consider this patient at high risk for Bethesda III nodules malignancy and recommend diagnostic lobectomy (**Figure 3A**).

To support clinical implementation of this predictive model, we developed a web-based calculator that enables physicians to input patient-specific clinical data and immediately obtain malignancy risk estimates for Bethesda III nodules, thereby supporting more informed clinical decisions. The tool streamlines risk assessment while facilitating personalized patient management through individualized risk stratification. The calculator is publicly accessible online at https://13583155338-l.shinyapps.io/PredictionofAUS/ (**Figure 3B**).

## 4 Discussion

According to the 2023 TBSRTC, the estimated malignant risk for Bethesda III nodules ranges from 10% to 30%, but recent studies report a significantly higher malignancy rate than predicted (5, 7, 33). More importantly, after being informed of the 10-30% malignancy risk and receiving follow-up treatment recommendations for US-FNA, these cases show higher rates of inconclusive results (up to 60%) on repeat FNA (34, 35). Although several studies have addressed this challenge, most focus on specific factors, with few providing comprehensive clinical, ultrasound, genetic, and pathological data. Additionally, many studies are limited by small sample sizes due to the absence of comprehensive postoperative pathological data, hindering their ability to conduct balanced research or only allowing for one-sided conclusions. Ahn, H. S. et al. (23) included surgical and repeat biopsy results to analyze nodule characteristics. By combining ultrasound features, nodule size, and cytological subtypes, they provided more accurate malignancy risk stratification for Bethesda III nodules. Ahmadi, S. et al. (36) observed 141 Bethesda III nodules with multigene testing and 12 months of ultrasound follow-up. The study showed a 100% negative predictive value, demonstrating the feasibility of follow-up using multigene testing for these nodules. Numerous studies have shown that multigene testing achieves a negative predictive value close to 100% in follow-up observation of Bethesda III nodules, proving its feasibility (37, 38). Therefore, we innovatively included Bethesda III nodules with surgical pathology, repeat biopsy, and multigene testing. We then compared logistic regression and five ML models to predict and analyze the malignancy risk of Bethesda III nodules, ultimately selecting logistic regression as the ideal model for developing predictive models and nomograms.

Due to the lack of standardized guidelines or consensus for feature selection in predictive models, it remains unclear how many features should be included in the model. Relevant risk variables often exhibit correlations, and multicollinearity can cause issues such as overfitting and computational complexity. To address this, we applied six feature selection methods to the study population, aiming to reduce the high correlation between predictors and capture the complex relationships with outcome variables.

We selected BRAF, composition, shape, orientation, and TI-RADS, with BRAF identified as the most significant predictor. The standalone BRAF V600E test has become a relatively cost-effective and routine preoperative tool in many tertiary hospitals across China. In this study, the odds ratio (OR) for BRAF was 17, and the BRAF V600E mutation rate in patients diagnosed with Bethesda III nodules and ultimately confirmed to have PTC reached 87.93% (39). However, BRAF for malignancy detection in the Bethesda III nodules population has a sensitivity of only 30%-40%. Research by Paspala, A. et al. (40) found that the detection rate of BRAF mutations in Bethesda III is lower than in Bethesda V/VI, suggesting its greater utility for stratification in high-risk subgroups but also its limitations in Bethesda III nodules. When BRAF is negative, gene testing cannot predict whether Bethesda III nodules are benign or malignant, with a negative predictive value of 38.1%-52.6% (39, 41). Therefore, malignancy prediction in Bethesda III nodules requires a combined approach that integrates ultrasound characteristics for improved diagnostic accuracy (42, 43). Composition, shape, and orientation are independent imaging risk factors for thyroid nodules and should be incorporated into predictive models for Bethesda III nodules malignancy. Jin, L. et al. (44) demonstrated that spatial heterogeneity in malignant nodules, quantified by ultrasound contrast enhancement, was significantly higher. Ultrasound findings of nodules with blurred margins, lobulated, or irregular contours suggest a higher likelihood of malignancy in Bethesda III nodules (45, 46). Thyroid nodule taller-than-wide (TTW) feature is a strong predictor of malignancy. Studies have shown that a shape with TTW yields a diagnostic accuracy of up to 83%, with a specificity of 73% (47). Additionally, combining composition, shape, orientation, and other ultrasound features significantly enhances both sensitivity and specificity in diagnosis (43, 48).

This study also included a subgroup analysis of Bethesda III nodules and examined the correlation between thyroid function and the benign or malignant nature of Bethesda III nodules. The findings show that the atypical nuclear category significantly differs from other categories in multiple aspects. First, the risk of malignancy (ROM) in cases with atypical nuclei is significantly higher than in cases with architectural atypia. Categorizing Bethesda III cases into nuclear atypia and architectural atypia subgroups may improve ROM stratification (49). Second, the proportion of malignant tumors in Bethesda III nodules increases with higher TI-RADS classifications, though no such difference is observed in FLUS (45). Moreover, both nuclear and architectural atypia subcategories in AUS exhibit high ROM, classifying them into high-risk groups (50, 51). Therefore, this study categorized pathological features into nuclear and architectural atypia. Unfortunately, neither cytological atypia subtype showed statistical significance in predicting malignancy in our analysis. This result is likely attributable to the limited sample size in the subgroups, particularly for nuclear atypia, which resulted in insufficient statistical power to detect a potential difference. The 2023 TBSRTC revision has streamlined subcategorization into two classifications: AUS-nuclear and AUS-other. This modification indirectly demonstrates the limitations of relying solely on traditional cytological subtypes for predicting malignancy risk, as subcategorization alone cannot provide sufficient clinical decision-making support. Future studies will seek to increase the sample size to investigate the relationship between pathological subtypes and the malignancy rate of Bethesda III nodules. The study found significant differences in TgAb levels between benign and malignant nodules, suggesting its potential role in preoperative malignancy diagnosis. Retrospective studies in Chinese patients showed that the TgAb positivity rate was significantly higher in PTC patients than in those with benign thyroid nodules, suggesting a potential link to malignant tumor occurrence (52).

In the era of big data, machine learning models for predicting clinical events have become increasingly important. Clinical EMR data is relatively objective, accurate, and easily accessible for clinicians and researchers. Combining EMR data with complex machine learning algorithms facilitates the development of clinical prediction models (53). This study demonstrates the capabilities of various machine learning models in predicting the benign or malignant nature of Bethesda III nodules and aims to identify the most suitable algorithm based on the dataset’s characteristics and the study’s objectives. Different machine learning algorithms have distinct principles, strengths, and applicable scenarios, but also limitations and biases. LR are traditional classification algorithms suited for linear relationships but sensitive to outliers. Decision trees, based on tree structures, are prone to overfitting and noise sensitivity. Random forests, using simple averaging or voting strategies, require significant computational resources and have poor interpretability. SVM optimize objective functions to find the optimal hyperplane but incur high computational costs and require careful parameter tuning. XGB, employing gradient boosting, requires long training times and extensive hyperparameter tuning. KNN are computationally intensive and struggle with high-dimensional data (54–56). In this study, the LR model achieved an AUC of 0.823 in the validation set, with superior sensitivity, recall, and F1 scores compared to more complex models, demonstrating its practical significance. When the primary goal is to analyze the relationship between outcomes and risk factors, especially with small datasets, traditional methods like logistic regression may be sufficient (57). This finding supports the argument that machine learning does not always outperform LR in predictive modelling (58). Additionally, the generalizability of the models, assessed through five-fold cross-validation, indicates that they can handle unseen data and reliably perform risk assessments in new clinical contexts. This comprehensive approach improved the predictive accuracy of the models and enhanced their reliability in practical applications.

The LR model outperformed others in discriminative ability, calibration, and clinical net benefit. The newly developed LR model, which integrates easily accessible pathological, ultrasound, and clinical features, performed well, with an AUC of 0.823 in the validation set. Besides AUC, we evaluated the LR model’s performance using other metrics, including precision, recall, F1 score, and Brier score. Due to its simple structure, the LR model excelled, demonstrating outstanding interpretability and high performance across these key metrics. The LR model’s higher F1 score reflects a reasonable balance between precision and recall, minimizing both false positives and false negatives, which is critical for clinical decision-making. The calibration plot showed good agreement between predicted and actual values. More importantly, decision curve analysis revealed that the LR model provides substantial clinical net benefit, supporting clinical decision-making. In terms of applicability, the LR model is characterized by its simple structure and high interpretability. However, the differences in predictive performance across models were not significant, likely due to the limited sample size of the validation set. Considering its interpretability, calibration, clinical utility, and potential for further analysis, the LR model remains the optimal choice. Additionally, incorporating AUS subtypes and thyroid function tests into the model may improve its predictive performance. The model’s low specificity inevitably increases false-positive rates in clinical settings. While these false positives may prompt additional diagnostic procedures, they rarely result in immediate radical interventions. Decision curve analysis confirms that despite this limitation, the model prevents more unnecessary procedures than it generates when benchmarked against universal treatment strategies. In addition, we recommend that future studies incorporate additional pathological and biochemical clinical data to potentially improve predictive outcomes.

This study developed a nomogram, which was evaluated through 500 bootstrap iterations and assessed for predictive performance using several goodness-of-fit tests, including the Hosmer–Lemeshow test, AUC-ROC, calibration curve, and DCA. The Hosmer–Lemeshow test showed good agreement between predicted and observed values (P = 0.414). The observed AUC difference (Δ = 0.025) between the training and validation cohorts indicates that the sample size is adequate to assess the model’s general applicability and effectiveness. The calibration curve also showed strong consistency between predicted and actual outcomes, underscoring the model’s reliability. Furthermore, the DCA results demonstrated that across a wide range of probability thresholds, the nomogram consistently offered greater clinical benefit than universal treatment or no treatment. Previous studies have also developed nomograms to predict the benign or malignant nature of Bethesda III nodules using systematic risk factors. Yoon, J. H. et al. (15) reported a nomogram with an AUC of 0.754 but did not evaluate its applicability using DCA. Öcal, B. et al. (59) reported an AUC of 0.784 in the validation set, although their dataset included uncertain nodules from Bethesda III, IV, and V categories. Zhong, L. et al. (16) described a nomogram with an AUC of 0.823, but its applicability was more limited, with a threshold probability range of 21% to 70%. Cao, Y. et al.0 (14) reported a nomogram with an AUC of 0.80, comparing two machine learning methods to traditional logistic regression. The nomogram developed in this study has an AUC higher than those reported in the aforementioned studies and shows broader applicability in DCA. Moreover, these previous studies employed only logistic regression or a few machine learning algorithms, without comparing them to a broader range of machine learning models. As a result, this study is more innovative and comprehensive. By using this nomogram, clinicians can accurately predict the benign or malignant nature of Bethesda III nodules, thus guiding clinical decision-making.

This study has several limitations. First, although robust internal validation through cross-validation and bootstrap methods strengthened the methodological rigor, the lack of external validation with an independent cohort remains a significant limitation. The single-center, retrospective nature of our dataset may introduce selection bias, and the small sample size could lead to overfitting in machine learning models, limiting the model’s generalizability across clinical practices with varying demographic characteristics, institutions, and regions. To address this limitation, future research should prioritize multicenter collaborations to validate and optimize the model in diverse settings, ensuring broader clinical applicability. Second, while our case selection included Bethesda III nodules patients followed up in outpatient settings without surgery, we did not conduct prospective follow-up for all patients. We used BRAF mutation analysis as part of routine preoperative testing, but did not include the 12-gene panel in the routine screening, which may have caused discrepancies in molecular analysis results. Patients who underwent surgery, repeat FNA, or molecular testing likely represent a higher-risk subset compared to those managed conservatively. Although the 12-gene test and repeat biopsy have nearly 100% negative predictive value, biases may still arise due to the relatively short follow-up period. Furthermore, we acknowledge that the minimum six-month follow-up period for non-surgical cases may be insufficient to capture all slow-growing malignancies. Future studies should prioritize long-term follow-up and include these additional mutations to enhance the comprehensiveness and accuracy of the predictive model. Third, our study cohort presented a malignancy rate of 65.2%, which is substantially higher than the 13-30% rate typically estimated for Bethesda III nodules. Consequently, our model was trained on a higher-risk population than is typically encountered in general clinical practice. Fourth, our study did not employ an exhaustive hyperparameter tuning process, such as grid search or random search, for the more complex machine learning models like Random Forest and XGBoost. Lastly, inter-observer variability in the interpretation of US and FNAB results may occur. In conclusion, of the six machine learning algorithms evaluated, LR demonstrated the best performance in this study.

Consequently, a logistic regression-based nomogram was developed to predict the benign or malignant nature of Bethesda III nodules. Future studies should focus on further prospective external validation to assess whether follow-up strategies based on the final predictive model can effectively predict the benign or malignant nature of Bethesda III nodules.

## Data Availability

The raw data supporting the conclusions of this article will be made available by the authors, without undue reservation.

## References

[1] Durante C Grani G Lamartina L Filetti S Mandel SJ Cooper DS. The diagnosis and management of thyroid nodules: A review. JAMA. (2018) 319:914–24. doi: 10.1001/jama.2018.0898, PMID: 29509871

[2] Grani G Sponziello M Filetti S Durante C. Thyroid nodules: Diagnosis and management. Nat Rev Endocrinol. (2024) 20:715–28. doi: 10.1038/s41574-024-01025-4, PMID: 39152228

[3] Ali SZ Baloch ZW Cochand-Priollet B Schmitt FC Vielh P VanderLaan PA. The 2023 Bethesda system for reporting thyroid cytopathology. Thyroid. (2023) 33:1039–44. doi: 10.1089/thy.2023.0141, PMID: 37427847

[4] Hassan I Hassan L Balalaa N Askar M Alshehhi H Almarzooqi M. The incidence of thyroid cancer in Bethesda III thyroid nodules: A retrospective analysis at a single endocrine surgery center. Diagnostics (Basel). (2024) 14, 1026. doi: 10.3390/diagnostics14101026, PMID: 38786324 PMC11119920

[5] Pasha HA Dhanani R Mughal A Ahmed KS Suhail A. Malignancy rate in thyroid nodules with atypia or follicular lesion of undetermined significance. Int Arch Otorhinolaryngol. (2020) 24:e221–26. doi: 10.1055/s-0039-1698784, PMID: 32256845 PMC6986942

[6] Jiragawasan C Himakhun W. The risk of Malignancy in the atypia of undetermined significance/follicular lesion of undetermined significance (AUS/FLUS) category subgroups: A Thai institute experience. J Am Soc Cytopathol. (2024) 13:16–22. doi: 10.1016/j.jasc.2023.09.006, PMID: 37903698

[7] Yoo WS Ahn HY Ahn HS Chung YJ Kim HS Cho BY. Malignancy rate of Bethesda category III thyroid nodules according to ultrasound risk stratification system and cytological subtype. Med (Baltimore). (2020) 99:e18780. doi: 10.1097/md.0000000000018780, PMID: 31914102 PMC6959967

[8] Raghunathan R Longstaff XR Hughes EG Li SJ Sant VR Tseng CH. Diagnostic performance of molecular testing in indeterminate (Bethesda III and IV) thyroid nodules with Hürthle cell cytology. Surgery. (2024) 175:221–27. doi: 10.1016/j.surg.2023.05.046, PMID: 37926582

[9] Geng J Wang H Liu Y Tai J Jin Y Zhang J. Correlation between BRAF (V600E) mutation and clinicopathological features in pediatric papillary thyroid carcinoma. Sci China Life Sci. (2017) 60:729–38. doi: 10.1007/s11427-017-9083-8, PMID: 28646474

[10] Fakhruddin N Jabbour M Novy M Tamim H Bahmad H Farhat F. BRAF and NRAS mutations in papillary thyroid carcinoma and concordance in BRAF mutations between primary and corresponding lymph node metastases. Sci Rep. (2017) 7:4666. doi: 10.1038/s41598-017-04948-3, PMID: 28680105 PMC5498648

[11] Sapio MR Posca D Troncone G Pettinato G Palombini L Rossi G. Detection of BRAF mutation in thyroid papillary carcinomas by mutant allele-specific PCR amplification (MASA). Eur J Endocrinol. (2006) 154:341–8. doi: 10.1530/eje.1.02072, PMID: 16452550

[12] Nikiforov YE Ohori NP Hodak SP Carty SE LeBeau SO Ferris RL. Impact of mutational testing on the diagnosis and management of patients with cytologically indeterminate thyroid nodules: A prospective analysis of 1056 FNA samples. J Clin Endocrinol Metab. (2011) 96:3390–7. doi: 10.1210/jc.2011-1469, PMID: 21880806 PMC3205883

[13] Patel KN Angell TE Babiarz J Barth NM Blevins T Duh QY. Performance of a genomic sequencing classifier for the preoperative diagnosis of cytologically indeterminate thyroid nodules. JAMA Surg. (2018) 153:817–24. doi: 10.1001/jamasurg.2018.1153, PMID: 29799911 PMC6583881

[14] Cao Y Yang Y Chen Y Luan M Hu Y Zhang L. Optimizing thyroid AUS nodules Malignancy prediction: A comprehensive study of logistic regression and machine learning models. Front Endocrinol (Lausanne). (2024) 15:1366687. doi: 10.3389/fendo.2024.1366687, PMID: 39568807 PMC11576180

[15] Yoon JH Lee HS Kim EK Moon HJ Kwak JY. A nomogram for predicting Malignancy in thyroid nodules diagnosed as atypia of undetermined significance/follicular lesions of undetermined significance on fine needle aspiration. Surgery. (2014) 155:1006–13. doi: 10.1016/j.surg.2013.12.035, PMID: 24630147

[16] Zhong L Shi L Lai J Hu Y Gu L. Combined model integrating clinical, radiomics, BRAF(V600E) and ultrasound for differentiating between benign and Malignant indeterminate cytology (Bethesda III) thyroid nodules: A bi-center retrospective study. Gland Surg. (2024) 13:1954–64. doi: 10.21037/gs-24-310, PMID: 39678423 PMC11635559

[17] Wu Y Rao K Liu J Han C Gong L Chong Y. Machine learning algorithms for the prediction of central lymph node metastasis in patients with papillary thyroid cancer. Front Endocrinol (Lausanne). (2020) 11:577537. doi: 10.3389/fendo.2020.577537, PMID: 33193092 PMC7609926

[18] Yao J Wang Y Lei Z Wang K Li X Zhou J. AI-generated content enhanced computer-aided diagnosis model for thyroid nodules: A chatGPT-style assistant. arXiv preprint arXiv. (2024) 2402:2401. doi: 10.48550/arXiv.2402.02401

[19] Yao J Wang Y Lei Z Wang K Feng N Dong F. Multimodal GPT model for assisting thyroid nodule diagnosis and management. NPJ Digit Med. (2025) 8:245. doi: 10.1038/s41746-025-01652-9, PMID: 40319170 PMC12049458

[20] Yao J Zhang Y Shen J Lei Z Xiong J Feng B. AI diagnosis of Bethesda category IV thyroid nodules. iScience. (2023) 26:108–7. doi: 10.1016/j.isci.2023.108114, PMID: 37867955 PMC10589877

[21] Gupta A Bajaj S Nema P Purohit A Kashaw V Soni V. Potential of AI and ML in oncology research including diagnosis, treatment and future directions: A comprehensive prospective. Comput Biol Med. (2025) 189:109918. doi: 10.1016/j.compbiomed.2025.109918, PMID: 40037170

[22] Collins GS Moons KGM Dhiman P Riley RD Beam AL Van Calster B. TRIPOD+AI statement: Updated guidance for reporting clinical prediction models that use regression or machine learning methods. BMJ. (2024) 385:e078378. doi: 10.1136/bmj-2023-078378, PMID: 38626948 PMC11019967

[23] Ahn HS Na DG Kim JH. Risk stratification of thyroid nodules diagnosed as Bethesda category III by ultrasound, size, and cytology. Korean J Radiol. (2024) 25:924–33. doi: 10.3348/kjr.2024.0292, PMID: 39344549 PMC11444854

[24] Zhou J Yin L Wei X Zhang S Song Y Luo B. 2020 Chinese guidelines for ultrasound Malignancy risk stratification of thyroid nodules: The C-TIRADS. Endocrine. (2020) 70:256–79. doi: 10.1007/s12020-020-02441-y, PMID: 32827126

[25] Vittinghoff E McCulloch CE. Relaxing the rule of ten events per variable in logistic and Cox regression. Am J Epidemiol. (2007) 165:710–8. doi: 10.1093/aje/kwk052, PMID: 17182981

[26] van Smeden M Moons KGM de Groot JA Collins GS Altman DG Eijkemans MJ. Sample size for binary logistic prediction models: Beyond events per variable criteria. Stat Methods Med Res. (2019) 28:2455–74. doi: 10.1177/0962280218784726, PMID: 29966490 PMC6710621

[27] Yuan X Xu Q Du F Gao X Guo J Zhang J. Development and validation of a model to predict cognitive impairment in traumatic brain injury patients: A prospective observational study. EClinicalMedicine. (2025) 80:103023. doi: 10.1016/j.eclinm.2024.103023, PMID: 39850016 PMC11753911

[28] Riley RD Ensor J Snell KIE Harrell FE Jr Martin GP Reitsma JB. Calculating the sample size required for developing a clinical prediction model. BMJ. (2020) 368:m441. doi: 10.1136/bmj.m441, PMID: 32188600

[29] Dong J Jin Z Li C Yang J Jiang Y Li Z. Machine learning models with prognostic implications for predicting gastrointestinal bleeding after coronary artery bypass grafting and guiding personalized medicine: Multicenter cohort study. J Med Internet Res. (2025) 27:e68509. doi: 10.2196/68509, PMID: 40053791 PMC11926454

[30] Xu Z Chen Q Zhou Z Sun J Tian G Liu C. Screening risk factors for the occurrence of wedge effects in intramedullary nail fixation for intertrochanteric fractures in older people via machine learning and constructing a prediction model: a retrospective study. BMC Musculoskelet Disord. (2025) 26:403. doi: 10.1186/s12891-025-08619-7, PMID: 40264104 PMC12016347

[31] Qi W Wang Y Wang Y Huang S Li C Jin H. Prediction of postpartum depression in women: development and validation of multiple machine learning models. J Transl Med. (2025) 23:291. doi: 10.1186/s12967-025-06289-6, PMID: 40055720 PMC11887113

[32] Lee S Gornitz N Xing EP Heckerman D Lippert C. Ensembles of lasso screening rules. IEEE Trans Pattern Anal Mach Intell. (2018) 40:2841–52. doi: 10.1109/tpami.2017.2765321, PMID: 29989981 PMC6925025

[33] Saoud C Bailey GE Graham AJ Maleki Z. The Bethesda system for reporting thyroid cytopathology in the African American population: A tertiary centre experience. Cytopathology. (2024) 35:715–23. doi: 10.1111/cyt.13426, PMID: 39075743

[34] Allen L Al Afif A Rigby MH Bullock MJ Trites J Taylor SM. The role of repeat fine needle aspiration in managing indeterminate thyroid nodules. J Otolaryngol Head Neck Surg. (2019) 48:16. doi: 10.1186/s40463-019-0338-7, PMID: 30894222 PMC6425601

[35] Papazian MR Dublin JC Patel KN Oweity T Jacobson AS Brandler TC. Repeat fine-needle aspiration with molecular analysis in management of indeterminate thyroid nodules. Otolaryngol Head Neck Surg. (2023) 168:738–44. doi: 10.1177/01945998221093527, PMID: 35412868

[36] Ahmadi S Kotwal A Bikas A Xiang P Goldner W Patel A. Outcomes of cytologically indeterminate thyroid nodules managed with genomic sequencing classifier. J Clin Endocrinol Metab. (2024) 109:e2231–39. doi: 10.1210/clinem/dgae112, PMID: 38415829

[37] Steward DL Carty SE Sippel RS Yang SP Sosa JA Sipos JA. Performance of a multigene genomic classifier in thyroid nodules with indeterminate cytology: A prospective blinded multicenter study. JAMA Oncol. (2019) 5:204–12. doi: 10.1001/jamaoncol.2018.4616, PMID: 30419129 PMC6439562

[38] Shrestha RT Evasovich MR Amin K Radulescu A Sanghvi TS Nelson AC. Correlation between histological diagnosis and mutational panel testing of thyroid nodules: A two-year institutional experience. Thyroid. (2016) 26:1068–76. doi: 10.1089/thy.2016.0048, PMID: 27283257 PMC4976225

[39] Lei L Hong Qing S Wei L Ma Mo Yang F Xiao Xiang Y. The combined use of fine-needle aspiration (FNA) and BRAF V600E gene testing: Can it increase the definitive diagnosis rate of nodules categorized as Bethesda III for papillary thyroid carcinoma? Am Surg. (2024) 90:3209–15. doi: 10.1177/00031348241265143, PMID: 39047144

[40] Paspala A Bompetsi G Paschou SA Charalambopoulos A Pikoulis E Peppa M. The value of preoperative molecular testing in the management of Bethesda V and Bethesda VI thyroid tumors. (Hormones (Athens). (2025) 24:217–29. doi: 10.1007/s42000-024-00597-0, PMID: 39225945

[41] Oh MY Choi HM Jang J Son H Park SS Song M. Small multi-gene DNA panel can aid in reducing the surgical resection rate and predicting the Malignancy risk of thyroid nodules. Endocrinol Metab (Seoul). (2024) 39:777–92. doi: 10.3803/EnM.2024.2034, PMID: 39397516 PMC11525692

[42] Alyusuf EY Alhmayin L Albasri E Enani J Altuwaijri H Alsomali N. Ultrasonographic predictors of thyroid cancer in Bethesda III and IV thyroid nodules. Front Endocrinol (Lausanne). (2024) 15:1326134. doi: 10.3389/fendo.2024.1326134, PMID: 38405143 PMC10884110

[43] Wu L Shu H Chen W Gao Y Yuan Y Li X. Diagnostic value of thyroid imaging reporting and data system combined with BRAF(V600E) mutation analysis in Bethesda categories III-V thyroid nodules. Sci Rep. (2022) 12:5934. doi: 10.1038/s41598-022-09822-5, PMID: 35395862 PMC8993851

[44] Jin L Xu C Xie X Li F Lv X Du L. An algorithm of image heterogeneity with contrast-enhanced ultrasound in differential diagnosis of solid thyroid nodules. Ultrasound Med Biol. (2017) 43:104–10. doi: 10.1016/j.ultrasmedbio.2016.05.011, PMID: 28029495

[45] Kim J Shin JH Oh YL Hahn SY Park KW. Approach to Bethesda System category III thyroid nodules according to US-risk stratification. Endocr J. (2022) 69:67–74. doi: 10.1507/endocrj.EJ21-0300, PMID: 34408101

[46] Lee S Shin JH Oh YL Hahn SY. Subcategorization of Bethesda System category III by ultrasonography. Thyroid. (2016) 26:836–42. doi: 10.1089/thy.2015.0637, PMID: 27094511

[47] Papapostolou KD Evangelopoulou CC Ioannidis IA Kassi GN Morfas KS Karaminas NI. Taller-than-wide thyroid nodules with microcalcifications are at high risk of Malignancy. In Vivo. (2020) 34:2101–05. doi: 10.21873/invivo.12014, PMID: 32606189 PMC7439909

[48] Alhajlan M Al-Masabi M Al Mansour M Saihb A AlAyed S Alwadai R. The accuracy of fine-needle aspiration cytology and ultrasonography in assessing thyroid nodules in correlation with histopathology: A retrospective study. Ann Med Surg (Lond). (2024) 86:7002–09. doi: 10.1097/ms9.0000000000002676, PMID: 39649848 PMC11623872

[49] Alden J Lambrou D Yang J. Two-tier subclassification of the Bethesda category III (Atypia of undetermined significance/follicular lesion of undetermined significance) in thyroid cytology. Diagn Cytopathol. (2024) 52:156–62. doi: 10.1002/dc.25261, PMID: 38095097

[50] Jin X Jing X Smola B Heider A. Malignant risk of pediatric Bethesda category III thyroid nodules subcategorized by nuclear atypia and other: A single institution experience. Cancer Cytopathol. (2024) 132:564–68. doi: 10.1002/cncy.22831, PMID: 38771850

[51] Bagis M Can N Sut N Tastekin E Erdogan EG Bulbul BY. A comprehensive approach to the thyroid Bethesda category III (AUS) in the transition zone between 2nd edition and 3rd edition of the Bethesda System for Reporting Thyroid Cytopathology: Subcategorization, nuclear scoring, and more. Endocr Pathol. (2024) 35:51–76. doi: 10.1007/s12022-024-09797-1, PMID: 38280141 PMC10944398

[52] Jia X Pang P Wang L Zhao L Jiang L Song Y. Clinical analysis of preoperative anti-thyroglobulin antibody in papillary thyroid cancer between 2011 and 2015 in Beijing, China: A retrospective study. Front Endocrinol (Lausanne). (2020) 11:452. doi: 10.3389/fendo.2020.00452, PMID: 32760349 PMC7373730

[53] Goecks J Jalili V Heiser LM Gray JW. How machine learning will transform biomedicine. Cell. (2020) 181:92–101. doi: 10.1016/j.cell.2020.03.022, PMID: 32243801 PMC7141410

[54] Motamedi F Pérez-Sánchez H Mehridehnavi A Fassihi A Ghasemi F. Accelerating big data analysis through lasso-random forest algorithm in QSAR studies. Bioinformatics. (2022) 38:469–75. doi: 10.1093/bioinformatics/btab659, PMID: 34979024

[55] Goin JE. Classification bias of the k-nearest neighbor algorithm. IEEE Trans Pattern Anal Mach Intell. (1984) 6:379–81. doi: 10.1109/tpami.1984.4767533, PMID: 21869207

[56] Rezvani S Wu J. Handling multi-class problem by intuitionistic fuzzy twin support vector machines based on relative density information. IEEE Trans Pattern Anal Mach Intell. (2023) 45:14653–64. doi: 10.1109/tpami.2023.3310908, PMID: 37651498

[57] Jiang W Li Z. Comparison of machine learning algorithms and nomogram construction for diabetic retinopathy prediction in type 2 diabetes mellitus patients. Ophthalmic Res. (2024) 67:537–48. doi: 10.1159/000541294, PMID: 39231456

[58] Christodoulou E Ma J Collins GS Steyerberg EW Verbakel JY Van Calster B. A systematic review shows no performance benefit of machine learning over logistic regression for clinical prediction models. J Clin Epidemiol. (2019) 110:12–22. doi: 10.1016/j.jclinepi.2019.02.004, PMID: 30763612

[59] Öcal B Korkmaz MH Yılmazer D Taşkın Türkmenoğlu T Bayır Ö Çadallı Tatar E. The Malignancy risk assessment of cytologically indeterminate thyroid nodules improves markedly by using a predictive model. Eur Thyroid J. (2019) 8:83–9. doi: 10.1159/000494720, PMID: 31192147 PMC6514482

