# Supplementary material for: Prediction of malignancy risk in Bethesda III nodules: development and validation of multiple machine learning models
Source: Front Endocrinol (Lausanne). 2025 Aug 29;16:1655828. doi: 10.3389/fendo.2025.1655828 (PMC12425777; doi:10.3389/fendo.2025.1655828)
Supplement: Supplementary file 1 [file SupplementaryFile1.pdf]

Supplementary materials

TRIPOD checklist 1

Table S1. The Distribution of Missing Values Across Multiple Variables 3

Table S2. The Calculation Process, Formula, and Results of Sample Size Determination. 4

Table S3. General characteristics of benign and malignant Bethesda III nodules. 5

Table S4. Univariate and Multivariate Logistic Regression Analysis in the Entire Cohort. 7

Table S5. Results of the multivariable logistic regression mode. 9

Table S6. Multicollinearity evaluation of predictor variables through variance inflation factor (VIF) analysis 9

Table S7. Delong test results of Bethesda III nodules differences between various machine learning models in the training set 10

Table S8. Delong test results of Bethesda III nodules differences between various machine learning models in the test set 10

Table S9. Parameters for six models 10

Table S10. Comparison of characteristics before and after multiple imputation in the entire set 11

Fig. S1. Flowchart of the study. 12

Fig. S2. Frequency and percentage of absent data among the predictor variables in the Entire Cohort. 14

Fig. S3. LASSO regression analysis plot. 15

Fig. S4. Variable shrinkage and selection using the Boruta algorithm. 16

Fig. S5. Random Forest machine variable importance plot. 17

Fig. S6. Forest plot of the logistic regression model. 18

Fig. S7. Decision tree visualization and variable importance plots 19

Fig. S8. Random forest variable importance plot 20

Fig. S9. XGBoost variable importance plot. 21

Fig. S10. Support vector machine variable importance plot. 22

Fig. S11. The confusion matrix of the six machine learning models in the training set. 23

Fig.S12. The confusion matrix of the six machine learning models in the test set. 24

Fig.S13. 5-fold CV of LR models in the training set and validation set. 25

Fig.S14. Performance of the logistic regression model among nomogram, BRAF, composition, shape, orientation and TI-RADS. 26

TRIPOD checklist

Section/Topic	Item	Development / evaluation[1]	Checklist item	Reported on page
**TITLE**				
Title	1	D; E	Identify the study as developing or evaluating the performance of a multivariable prediction model, the target population, and the outcome to be predicted	YES
**ABSTRACT**				
Abstract	2	D; E	See TRIPOD+AI for Abstracts checklist	YES
**INTRODUCTION**				
Background	3a	D; E	Explain the healthcare context (including whether diagnostic or prognostic) and rationale for developing or evaluating the prediction model, including references to existing models	YES
	3b	D; E	Describe the target population and the intended purpose of the prediction model in the context of the care pathway, including its intended users (e.g., healthcare professionals, patients, public)	YES
	3c	D; E	Describe any known health inequalities between sociodemographic groups	YES
Objectives	4	D; E	Specify the study objectives, including whether the study describes the development or validation of a prediction model (or both)	YES
**METHODS**				
Data	5a	D; E	Describe the sources of data separately for the development and evaluation datasets (e.g., randomised trial, cohort, routine care, or registry data), the rationale for using these data, and the representativeness of the data	YES
	5b	D; E	Specify the dates of the collected participant data, including the start and end of participant accrual and, if applicable, the end of follow-up	YES
Participants	6a	D; E	Specify key elements of the study setting (e.g., primary care, secondary care, general population), including the number and location of centers	YES
	6b	D; E	Describe the eligibility criteria for study participants	YES
	6c	D; E	Give details of any treatments received and how they were handled during model development or evaluation, if relevant	Not applicable
Data preparation	7	D; E	Describe any data pre-processing and quality checking, including whether this was similar across relevant sociodemographic groups	YES
Outcome	8a	D; E	Clearly define the outcome that is being predicted and the time horizon, including how and when assessed, the rationale for choosing this outcome, and whether the method of outcome assessment is consistent across sociodemographic groups	YES
	8b	D; E	If outcome assessment requires subjective interpretation, describe the qualifications and demographic characteristics of the outcome assessors	YES
	8c	D; E	Report any actions to blind assessment of the outcome to be predicted	Not applicable
Predictors	9a	D	Describe the choice of initial predictors (e.g., literature, previous models, all available predictors) and any pre-selection of predictors before model building	YES
	9b	D; E	Clearly define all predictors, including how and when they were measured (and any actions to blind assessment of predictors for the outcome and other predictors)	YES
	9c	D; E	If predictor measurement requires subjective interpretation, describe the qualifications and demographic characteristics of the predictor assessors	Not applicable
Sample size	10	D; E	Explain how the study size was arrived at (separately for development and evaluation), and justify that the study size was sufficient to answer the research question. Include details of any sample size calculation	YES
Missing data	11	D; E	Describe how missing data were handled. Provide reasons for omitting any data	YES
Analytical methods	12a	D	Describe how the data were used (e.g., for development and evaluation of model performance) in the analysis, including whether the data were partitioned, considering any sample size requirements	YES
	12b	D	Depending on the type of model, describe how predictors were handled in the analyses (functional form, rescaling, transformation, or any standardization).	YES
	12c	D	Specify the type of model, rationale[2], all model-building steps, including any hyperparameter tuning, and method for internal validation	YES
	12d	D; E	Describe if and how any heterogeneity in estimates of model parameter values and model performance was handled and quantified across clusters (e.g., hospitals, countries). See TRIPOD-Cluster foradditional considerations[3]	Not applicable
	12e	D; E	Specify all measures and plots used (and their rationale) to evaluate model performance (e.g., discrimination, calibration, clinical utility) and, if relevant, to compare multiple models	YES
	12f	E	Describe any model updating (e.g., recalibration) arising from the model evaluation, either overall or for particular sociodemographic groups or settings	Not applicable
	12g	E	For model evaluation, describe how the model predictions were calculated (e.g., formula, code, object, application programming interface)	YES
Class imbalance	13	D; E	If class imbalance methods were used, state why and how this was done, and any subsequent methods to recalibrate the model or the model predictions	Not applicable
Fairness	14	D; E	Describe any approaches that were used to address model fairness and their rationale	Not applicable
Model output	15	D	Specify the output of the prediction model (e.g., probabilities, classification). Provide details and rationale for any classification and how the thresholds were identified	YES

Training versus evaluation	16	D; E	Identify any differences between the development and evaluation data in a healthcare setting, eligibility criteria, outcome, and predictors	YES
Ethical approval	17	D; E	Name the institutional research board or ethics committee that approved the study and describe the participant-informed consent or the ethics committee waiver of informed consent	YES
**OPEN SCIENCE**				
Funding	18a	D; E	Give the source of funding and the role of the funders for the present study	YES
Conflicts of interest	18b	D; E	Declare any conflicts of interest and financial disclosures for all authors	YES
Protocol	18c	D; E	Indicate where the study protocol can be accessed or state that a protocol was not prepared	Not prepared
Registration	18d	D; E	Provide registration information for the study, including register name and registration number, or state that the study was not registered	Not registered
Data sharing	18e	D; E	Provide details of the availability of the study data	YES
Code sharing	18f	D; E	Provide details of the availability of the analytical code[4]	YES
**PATIENT & PUBLIC INVOLVEMENT**				
Patient & Public Involvement	19	D; E	Provide details of any patient and public involvement during the design, conduct, reporting, interpretation, or dissemination of the study or state no involvement.	No involvement
**RESULTS**				
Participants	20a	D; E	Describe the flow of participants through the study, including the number of participants with and without the outcome and, if applicable, a summary of the follow-up time. A diagram may be helpful.	YES
	20b	D; E	Report the characteristics overall and, where applicable, for each data source or setting, including the key dates, key predictors (including demographics), treatments received, sample size, number of outcome events, follow-up time, and amount of missing data. A table may be helpful. Report any differences across key demographic groups.	YES
	20c	E	For model evaluation, show a comparison with the development data of the distribution of important predictors (demographics, predictors, and outcome).	YES
Model development	21	D; E	Specify the number of participants and outcome events in each analysis (e.g., for model development, hyperparameter tuning, model evaluation)	YES
Model specification	22	D	Provide details of the full prediction model (e.g., formula, code, object, application programming interface) to allow predictions in new individuals and to enable third-party evaluation and implementation, including any restrictions to access or re-use (e.g., freely available, proprietary)[5]	YES
Model performance	23a	D; E	Report model performance estimates with confidence intervals, including for any key subgroups (e.g., sociodemographic). Consider plots to aid the presentation.	YES
	23b	D; E	If examined, report results of any heterogeneity in model performance across clusters. See TRIPOD Cluster for additional details[3].	Not applicable
Model updating	24	E	Report the results from any model updating, including the updated model and subsequent performance	Not applicable
**DISCUSSION**				
Interpretation	25	D; E	Give an overall interpretation of the main results, including issues of fairness in the context of the objectives and previous studies	YES
Limitations	26	D; E	Discuss any limitations of the study (such as a non-representative sample, sample size, overfitting, or missing data) and their effects on any biases, statistical uncertainty, and generalizability	YES
Usability of the model in the context of current care	27a	D	Describe how poor quality or unavailable input data (e.g., predictor values) should be assessed and handled when implementing the prediction model	YES
	27b	D	Specify whether users will be required to interact in the handling of the input data or use of the model and what level of expertise is required of users	YES
	27c	D; E	Discuss any next steps for future research, with a specific view to the applicability and generalizability of the model	YES

1 D=items relevant only to the development of a prediction model; E=items relating solely to the evaluation of a prediction model; D; E=items applicable to both the development and evaluation of a prediction model

2 Separately for all model building approaches.

3 TRIPOD-Cluster is a checklist of reporting recommendations for studies developing or validating models that explicitly account for clustering or explore heterogeneity in model performance (e.g., at different hospitals or centers). Debray et al, BMJ 2023; 380: e071018 [DOI: 10.1136/bmj-2022-071018]

4 This relates to the analysis code, for example, any data cleaning, feature engineering, model building, and evaluation.

5 This relates to the code to implement the model to get estimates of risk for a new individual.

From: Collins GS, Moons KGM, Dhiman P, et al. BMJ 2024;385:e078378. doi:10.1136/bmj-2023-078378

Table S1. The Distribution of Missing Values Across Multiple Variables

Variable	Total Entries	Missing Values	Missing Percentage
TSH	8004	44	0.55%
FT3	8004	44	0.55%
FT4	8004	44	0.55%
TgAb	8004	54	0.67%
TPOAb	8004	49	0.61%
Total	8804	235	2.94%

Abbreviations: TSH, Thyroid-Stimulating Hormone; FT4, Serum-free Thyroxine; FT3, Serum-free Triiodothyronine; TgAb, Thyroglobulin Antibody; TPOAb, Thyroid Peroxidase Antibody.

Table S2. The Calculation Process, Formula, and Results of Sample Size Determination.

Method	Sample Size(n)	Formula
**Rule of Thumb**		
10 Events Per Candidate Predictor Parameter	90	Each continuous candidate variable corresponds to one β coefficient. For categorical variables, the number of β coefficients is equal to the number of categories minus one. The required number of positive events is calculated as the total number of β coefficients summed and then multiplied by 10.
**Four-Step Procedure**		
Sample Size for Precise Outcome Risk or Mean Estimates	57	$n = \left(\frac{1.96}{\delta}\right)^2 \hat{\phi}(1 - \hat{\phi})$
Sample Size for Minimizing Prediction Error Across Individuals	96	$n = exp\left(\frac{-0.508 + 0.259In(\phi) + 0.504In(P) - In(MAPE)}{0.544}\right)$
Sample Size for Reducing Predictor Effect Shrinkage	196	$n = \frac{P}{(S-1)ln\left(1 - \frac{R_{cs}^2}{S}\right)}$
Sample Size for Minimizing Optimism in Model Fit	120	$R^2\ Nagelkehe = \frac{R_{cs}^2}{\max(R_{cs}^2)}, \max(R_{cs}^2) = 1 - \exp\left(\frac{2\ln L_{null}}{n}\right)$ $\ln L_{null} = Eln\left(\frac{E}{n}\right) + (n-E)\ln\left(1 - \frac{E}{n}\right), S = \frac{R_{cs}^2}{R_{cs}^2 + \delta\max(R_{cs}^2)}$ $n = \frac{P}{(S-1)\ln\left(1 - \frac{R_{cs}^2}{S}\right)}$

We identified a total of 29 candidate variables, with 5 predictive variables selected through six feature selection methods. According to the 2023 TBSRTC, the expected proportion of the endpoint event was estimated at 0.15. Based on these parameters, we calculated the sample size using the formula mentioned above.

For ease of application, we utilized and organized the pmsampsize package developed by Riley et al. in R to perform the calculations. The parameters of the above formula are explained as follows:

ϕ: Expected proportion of the endpoint event

δ: Absolute error range

P: Number of predictive variables

MAPE: Mean absolute prediction error

S: Shrinkage factor

R_{cs}^2: Cox-Snell pseudo R-squared

R^2 Nagelkerke: Nagelkerke R-squared, an adjusted version of Cox-Snell R-squared

Table S3. General characteristics of benign and malignant Bethesda III nodules.

Characteristics	Total N=276	Benign N=96	Malignance N=180	P value
Gender(%)				0.617
Male	60 (21.74)	23 (23.96)	37 (20.56)	
Female	216 (78.26)	73 (76.04)	143 (79.44)	
Age(median [IQR])	51.00 [41.00;57.25]	52.00 [45.00;60.25]	49.00 [39.75;57.00]	0.047
Nuclear atypia(%)				0.062
Absent	36 (13.04)	18 (18.75)	18 (10.00)	
Present	240 (86.96)	78 (81.25)	162 (90.00)	
Architectural atypia(%)				0.094
Present	25 (9.06)	13 (13.54)	12 (6.67)	
Absent	251 (90.94)	83 (86.46)	168 (93.33)	
$BRAF^{V600E}$ (%)				<0.001
Negative	163 (59.06)	86 (89.58)	77 (42.78)	
Positive	113 (40.94)	10 (10.42)	103 (57.22)	
Diameter (median [IQR])	5.20 [4.00;7.70]	6.85 [4.88;13.50]	5.00 [3.80;6.30]	<0.001
Solid composition(%)				<0.001
NO	40 (14.49)	31 (32.29)	9 (5.00)	
YES	236 (85.51)	65 (67.71)	171 (95.00)	
Nodule position 1(%)				0.392
Left lobe	134 (48.55)	51 (53.12)	83 (46.11)	
Right lobe	133 (48.19)	41 (42.71)	92 (51.11)	
Isthmus	9 (3.26)	4 (4.17)	5 (2.78)	
Marked Hypoechoic(%)				0.013
YES	255 (92.39)	83 (86.46)	172 (95.56)	
NO	21 (7.61)	13 (13.54)	8 (4.44)	
Unclear boundary(%)				<0.001
YES	49 (17.75)	29 (30.21)	20 (11.11)	
NO	227 (82.25)	67 (69.79)	160 (88.89)	
Irregular shape(%)				<0.001
YES	58 (21.01)	34 (35.42)	24 (13.33)	
NO	218 (78.99)	62 (64.58)	156 (86.67)	
Microcalcification(%)				0.097
YES	44 (15.94)	10 (10.42)	34 (18.89)	
NO	232 (84.06)	86 (89.58)	146 (81.11)	
Calcification(%)				0.841
No calcification	159 (57.61)	54 (56.25)	105 (58.33)	
Microcalcification	69 (25.00)	26 (27.08)	43 (23.89)	
Macrocalcification	48 (17.39)	16 (16.67)	32 (17.78)	
Halo(%)				0.085
Absent	244 (88.41)	80 (83.33)	164 (91.11)	
Present	32 (11.59)	16 (16.67)	16 (8.89)	
Orientation(%)				<0.001
Taller-than-wide	129 (46.74)	64 (66.67)	65 (36.11)	

Wider-than-tall	147 (53.26)	32 (33.33)	115 (63.89)	
Upper region(%)				0.254
YES	58 (21.01)	16 (16.67)	42 (23.33)	
NO	218 (78.99)	80 (83.33)	138 (76.67)	
Nodule Position 2(%)				0.455
Upper region	58 (21.01)	16 (16.67)	42 (23.33)	
Middle region	135 (48.91)	50 (52.08)	85 (47.22)	
Lower region	73 (26.45)	25 (26.04)	48 (26.67)	
Isthmus	10 (3.62)	5 (5.21)	5 (2.78)	
CDFI pattern (%)				0.003
Absent	211 (76.45)	63 (65.62)	148 (82.22)	
Present	65 (23.55)	33 (34.38)	32 (17.78)	
Echotexture(%)				0.226
Homogeneous	98 (35.51)	29 (30.21)	69 (38.33)	
Heterogeneous	178 (64.49)	67 (69.79)	111 (61.67)	
Posterior features(%)				0.354
Present	65 (23.55)	19 (19.79)	46 (25.56)	
Absent	211 (76.45)	77 (80.21)	134 (74.44)	
Shadowing Posterior features(%)				0.868
NO	201 (72.83)	71 (73.96)	130 (72.22)	
YES	75 (27.17)	25 (26.04)	50 (27.78)	
Suspicious LN (%)				0.652
YES	100 (36.23)	37 (38.54)	63 (35.00)	
NO	176 (63.77)	59 (61.46)	117 (65.00)	
TI-RADS(%)				<0.001
3	53 (19.20)	37 (38.54)	16 (8.89)	
4a	102 (36.96)	42 (43.75)	60 (33.33)	
4b	78 (28.26)	12 (12.50)	66 (36.67)	
4c	29 (10.51)	4 (4.17)	25 (13.89)	
5	14 (5.07)	1 (1.04)	13 (7.22)	
Solitary nodule(%)				0.691
YES	112 (40.58)	41 (42.71)	71 (39.44)	
NO	164 (59.42)	55 (57.29)	109 (60.56)	
TSH(median [IQR])	1.76 [1.08;2.58]	1.78 [1.05;2.30]	1.74 [1.09;2.70]	0.336
FT4(median [IQR])	13.58 [11.63;16.24]	14.30 [11.95;16.90]	13.30 [11.63;15.53]	0.045
FT3(median [IQR])	4.49 [4.07;5.01]	4.54 [4.04;5.04]	4.46 [4.08;5.00]	0.767
TgAb(median [IQR])	7.61 [1.15;19.88]	11.65 [1.80;19.30]	2.34 [1.00;20.30]	0.070
TPOAb(median [IQR])	7.08 [1.00;14.98]	9.00 [1.21;14.98]	2.62 [1.00;14.90]	0.103

Abbreviations: CDFI, Color Doppler Flow Imaging; LN, Lymph Node; TI-RADS, Thyroid Imaging Reporting and Data System; TSH, Thyroid-Stimulating Hormone; FT4, Serum-free Thyroxine; FT3, Serum-free Triiodothyronine; TgAb, Thyroglobulin Antibody; TPOAb, Thyroid Peroxidase Antibody.

Table S4. Univariate and Multivariate Logistic Regression Analysis in the Entire Cohort.

Characteristics	SR-FS		SR-BS & SR-BE	
	OR (95% CI)	*P-value*	OR (95% CI)	*P-value*
Gender				
Age				
Nuclear atypia				
Absent			Ref	
Present			1.865 (0.524-6.719)	0.333
Architectural atypia				
Present				
Absent				
BRAF^{V600E}				
Negative	Ref		Ref	
Positive	16.18 (5.857-54.01)	0	20.60 (6.660-85.21)	0
Diameter			1.026 (0.966-1.084)	0.346
Solid composition				
YES	Ref		Ref	
NO	2.846 (0.918-9.381)	0.074	3.886 (0.999-16.45)	0.054
Nodule position 1				
Left lobe				
Right lobe				
Isthmus				
Marked Hypoechoic				
NO			Ref	
YES			0.273 (0.041-1.651)	0.162
Unclear boundary				
NO			Ref	
YES			1.171 (0.274-4.933)	0.828
Irregular shape				
NO	Ref		Ref	
YES	2.851 (1.073-7.876)	0.037	2.428 (0.641-9.459)	0.191
Microcalcification				
YES				
NO				
Calcification				
No calcification				
Microcalcification				
Macrocalcification				
Halo				
Absent				
Present				
Orientation				
Wider-than-tall	Ref		Ref	
Taller-than-wide	2.411 (1.024-5.766)	0.045	2.384 (0.994-5.812)	0.052

Upper region				
YES				
NO				
Nodule Position 2				
Upper region				
Middle region				
Lower region				
Isthmus				
CDFI pattern				
Absent				
Present				
Echotexture				
Homogeneous				
Heterogeneous				
Posterior features				
Present				
Absent				
Shadowing Posterior				
NO				
YES				
Suspicious LN				
YES				
NO	Ref		Ref	
TI-RADS	2.193 (1.375-3.658)	0.002	2.150 (1.305-3.718)	0.004
Solitary nodule				
YES				
NO				
TSH				
FT4				
FT3	Ref		Ref	
TgAb	0.998 (0.995-1.000)	0.131	0.998 (0.995-1.000)	0.146
TPOAb				

Abbreviations: Ref, reference; CDFI, Color Doppler Flow Imaging; LN, Lymph Node; TI-RADS, Thyroid Imaging Reporting and Data System; TSH, Thyroid-Stimulating Hormone; FT4, Serum-free Thyroxine; FT3, Serum-free Triiodothyronine; TgAb, Thyroglobulin Antibody; TPOAb, Thyroid Peroxidase Antibody.

Table S5. Results of the multivariable logistic regression mode.

Characteristics	Univariable analysis		Multivariable analysis				
	OR (95% CI)	*P*-value	B	SE	Z-value	*P*-value	OR (95% CI)
BRAF^{V600E}							
Negative	Ref		Ref				Ref
Positive	14.00(6.043-38.39)	0	2.884	0.561	5.145	0	17.88 (6.480-59.67)
Solid composition							
YES	Ref		Ref				Ref
NO	8.233(3.571-20.86)	0	1.048	0.571	1.833	0.067	2.850 (0.948-9.157)
Irregular shape							
NO	Ref		Ref				Ref
YES	3.571(1.78-7.308)	0	0.993	0.505	1.968	0.049	2.699 (1.014-7.478)
Orientation							
Wider-than-tall	Ref		Ref				Ref
Taller-than-wide	3.812(2.056-7.242)	0	0.962	0.429	2.246	0.025	2.618 (1.137-6.155)
TI-RADS	2.84(1.983-4.244)	0	0.789	0.244	3.228	0.001	2.200 (1.387-3.638)

Abbreviations: Ref, reference; TI-RADS, Thyroid Imaging Reporting and Data System.

Table S6. Multicollinearity evaluation of predictor variables through variance inflation factor (VIF) analysis

Feature	VIF
BRAF^{V600E}	1.185
Solid composition	1.026
Irregular shape	1.051
Orientation	1.146
TI-RADS	1.113

Abbreviations: TI-RADS, Thyroid Imaging Reporting and Data System.

Table S7. Delong test results of Bethesda III nodules differences between various machine learning models in the training set

		DT	LR	RF	XGB	SVM	KNN
		**0.829**	**0.885**	**0.923**	**0.902**	**0.878**	**0.900**
**DT**	**0.829**	1	0.144	＜0.001	＜0.001	0.023	0.016
**LR**	**0.885**	0.144	1	0.220	0.617	0.832	0.653
**RF**	**0.923**	＜0.001	0.220	1	0.060	0.005	0.152
**XGB**	**0.902**	＜0.001	0.617	0.060	1	0.012	0.933
**SVM**	**0.878**	0.023	0.832	0.005	0.012	1	0.330
**KNN**	**0.900**	0.016	0.653	0.152	0.933	0.330	1

Abbreviations: LR, logistic regression; DT, decision tree; RF, random forest; XGBoost, extreme gradient boosting; SVM, support vector machine; KNN, k-nearest neighbours; AUC, area under the receiver-operating curve.

Table S8. Delong test results of Bethesda III nodules differences between various machine learning models in the test set

		DT	LR	RF	XGB	SVM	KNN
		**0.758**	**0.823**	**0.792**	**0.817**	**0.822**	**0.772**
**DT**	0.758	1	0.351	0.333	0.078	0.128	0.793
**LR**	0.823	0.351	1	0.645	0.925	0.977	0.454
**RF**	0.792	0.333	0.645	1	0.162	0.223	0.401
**XGB**	0.817	0.078	0.925	0.162	1	0.800	0.156
**SVM**	0.822	0.128	0.977	0.223	0.800	1	0.193
**KNN**	0.772	0.793	0.454	0.401	0.156	0.193	1

Abbreviations: LR, logistic regression; DT, decision tree; RF, random forest; XGBoost, extreme gradient boosting; SVM, support vector machine; KNN, k-nearest neighbours; AUC, area under the receiver-operating curve.

Table S9. Parameters for six models

**Model**	**Parameter**
Logistic Regression (LR)	family = binomial(logit), tol = 1e-8, maxit = 25
Decision Tree (DT)	cp = 0.01
Random Forest (RF)	ntree= 500, mtry = 3
Support Vector Machine (SVM)	kernel= "linear", C = 1
K-Nearest Neighbors (KNN)	k = 3
Extreme Gradient Boosting (XGBoost)	eta = 0.3, max_depth = 2, subsample=0.7, colsample_bytree = 0.4 gamma =0.001

Table S10. Comparison of characteristics before and after multiple imputation in the entire set

Characteristics	**Before multiple imputation**	**After multiple imputation**	P value
Gender(%)			＞0.99
Age(median [IQR])			＞0.99
Nuclear atypia(%)			＞0.99
Architectural atypia(%)			＞0.99
BRAF^{V600E} (%)			＞0.99
Diameter (median [IQR])			＞0.99
Solid composition(%)			＞0.99
Nodule position 1(%)			＞0.99
Marked Hypoechoic(%)			＞0.99
Unclear boundary(%)			＞0.99
Irregular shape(%)			＞0.99
Microcalcification(%)			＞0.99
Calcification(%)			＞0.99
Halo(%)			＞0.99
Orientation(%)			＞0.99
Upper region(%)			＞0.99
Nodule Position 2(%)			＞0.99
CDFI pattern (%)			＞0.99
Echotexture(%)			＞0.99
Posterior features(%)			＞0.99
Shadowing Posterior features(%)			＞0.99
Suspicious LN (%)			＞0.99
TI-RADS(%)			＞0.99
Solitary nodule(%)			＞0.99
TSH(median [IQR])	1.76 [1.08;2.58]	1.77 [1.11;2.65]	0.618
FT4(median [IQR])	13.58 [11.63;16.24]	13.64 [11.72;16.12]	0.828
FT3(median [IQR])	4.49 [4.07;5.01]	4.50 [4.08;5.01]	0.848
TgAb(median [IQR])	7.61 [1.15;19.88]	7.61 [1.15;20.25]	0.842
TPOAb(median [IQR])	7.08 [1.00;14.98]	7.43 [1.00;15.35]	0.942

Fig. S1. Flowchart of the study.

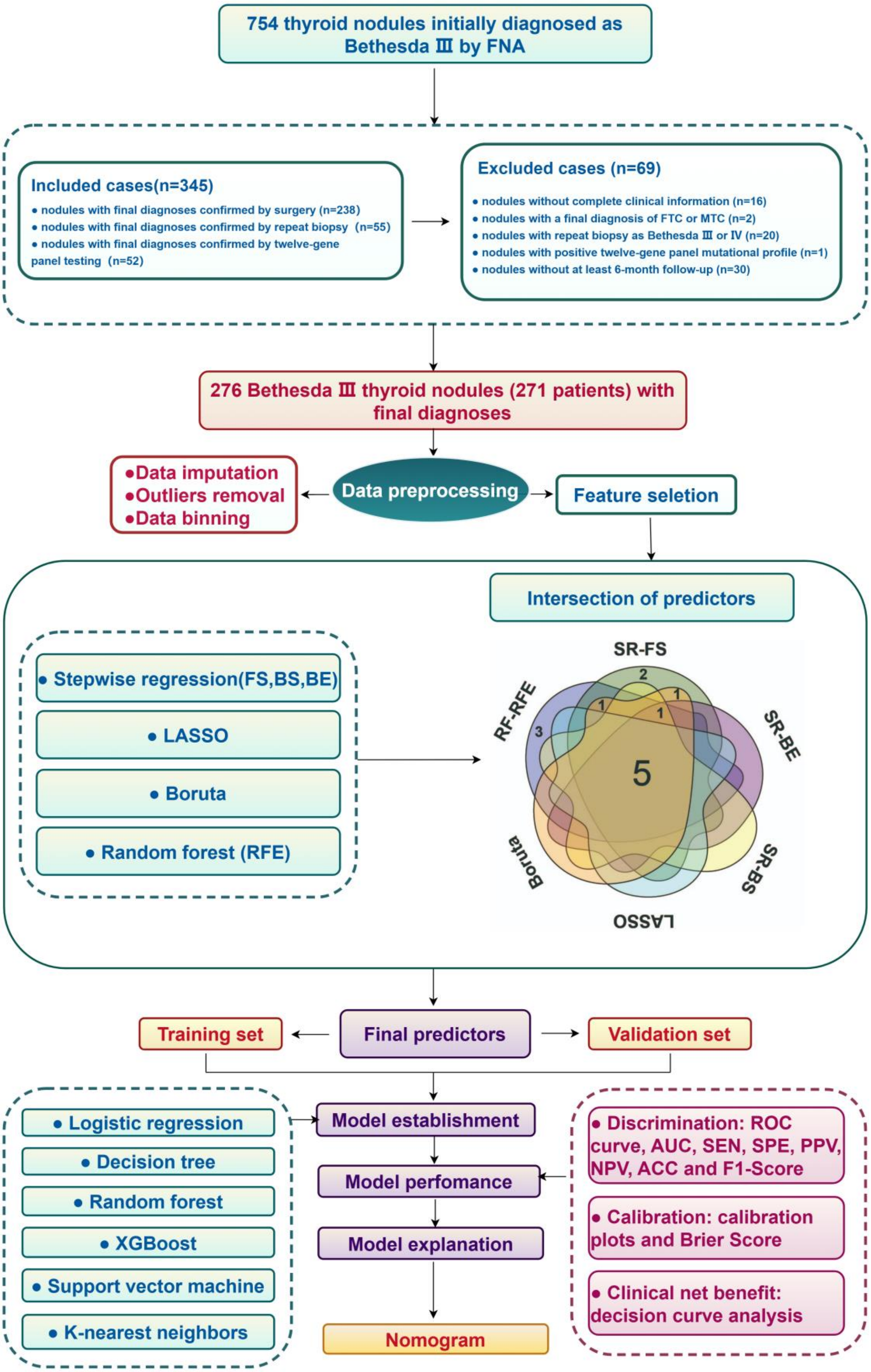

Abbreviations: LR, logistic regression; DT, decision tree; RF, random forest; XGBoost, extreme gradient boosting; SVM, support vector machine; KNN, k-nearest neighbours; AUC, area under the receiver-operating curve. SR-FS stepwise regression-forward selection, SR-BS stepwise regression-backward selection, SR-BE stepwise regression-bidirectional elimination, LASSO least absolute shrinkage and selection operator, RF-RFE random forest -recursive feature elimination; AUC, area under curve; SEN, sensitivity; SPE, specificity; PPV, positive predictive value; NPV, negative predictive value; ACC, accuracy.

Fig. S2. Frequency and percentage of absent data among the predictor variables in the Entire Cohort.

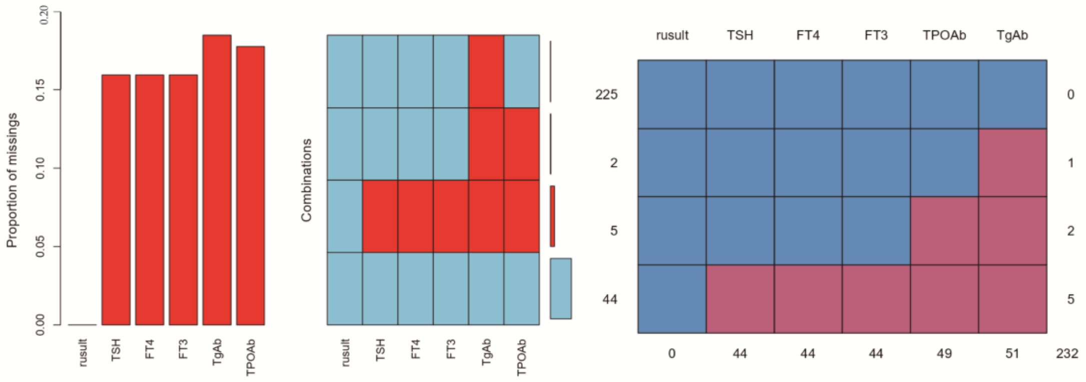

Abbreviations: TSH, Thyroid-Stimulating Hormone; FT4, Serum-free Thyroxine; FT3, Serum-free Triiodothyronine; TgAb, Thyroglobulin Antibody; TPOAb, Thyroid Peroxidase Antibody.

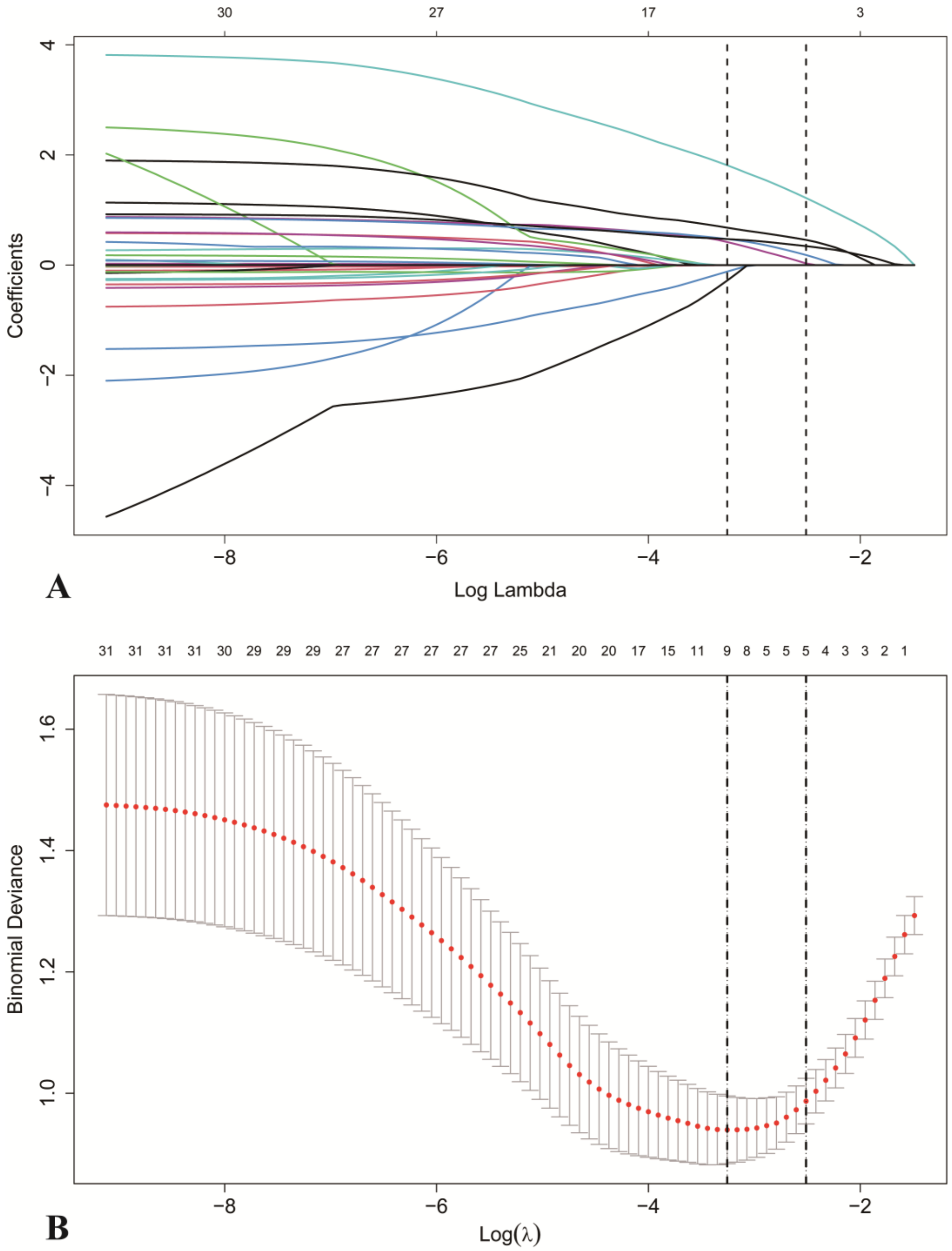

Fig. S3. LASSO regression analysis plot. A Regression coefficient plot for predictor variables with different penalty parameters (λ). **B** Cross-validation plot for penalty parameters (λ).

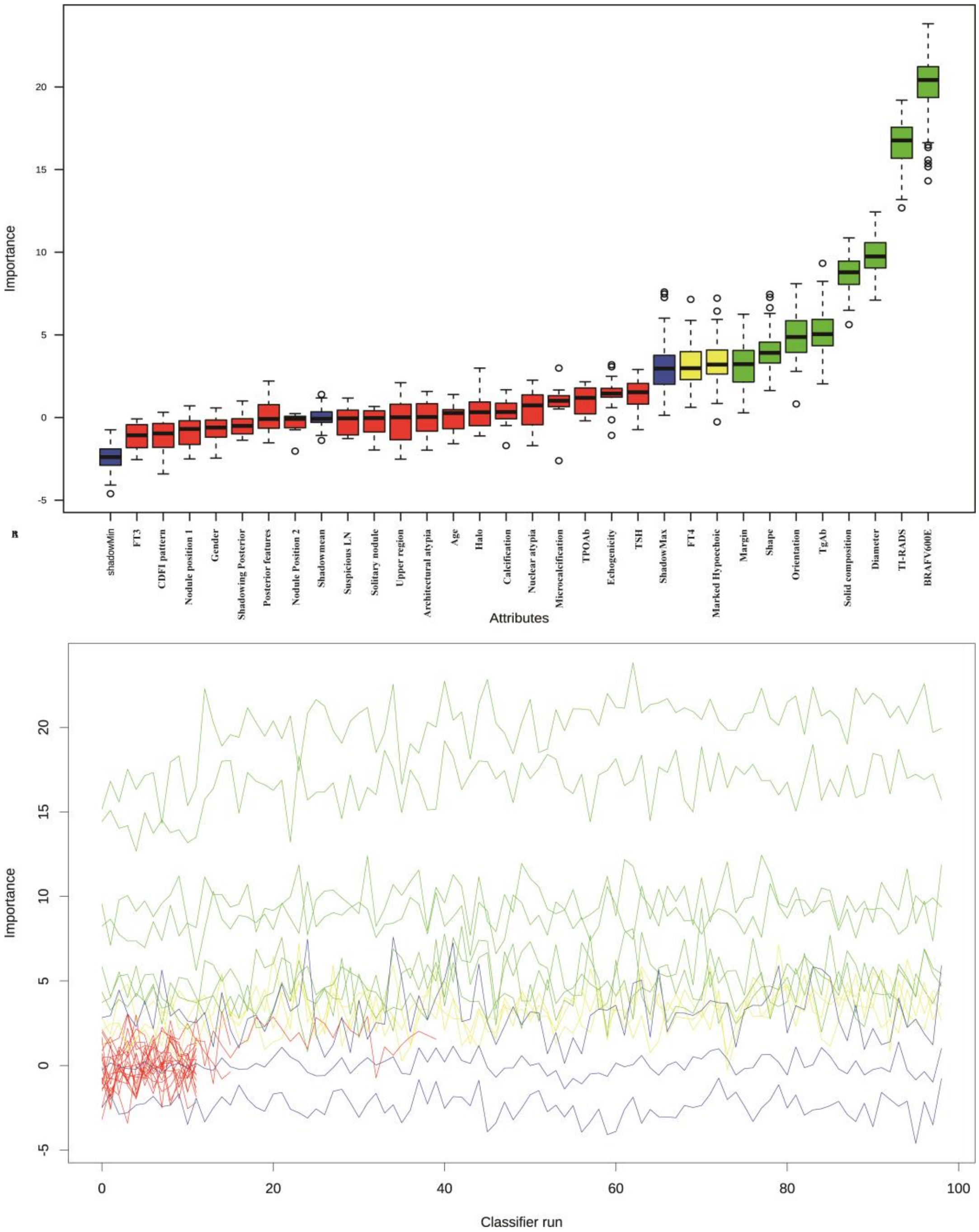

Fig. S4. Variable shrinkage and selection using the Boruta algorithm. A Classifier run of Boruta analysis. Every curve represents the Z-value of each variable in the model calculation. **B** Boruta feature selection model identified 8 of the 29 variables that demonstrated a strong association with malignant Bethesda Ⅲ nodules. The horizontal axis displays the number of variables, while the vertical axis shows the root mean square error value of each variable.

Abbreviations: CDFI, Color Doppler Flow Imaging; LN, Lymph Node; TI-RADS, Thyroid Imaging Reporting and Data System; TSH, Thyroid-Stimulating Hormone; FT4, Serum-free Thyroxine; FT3, Serum-free Triiodothyronine; TgAb, Thyroglobulin Antibody; TPOAb, Thyroid Peroxidase Antibody.

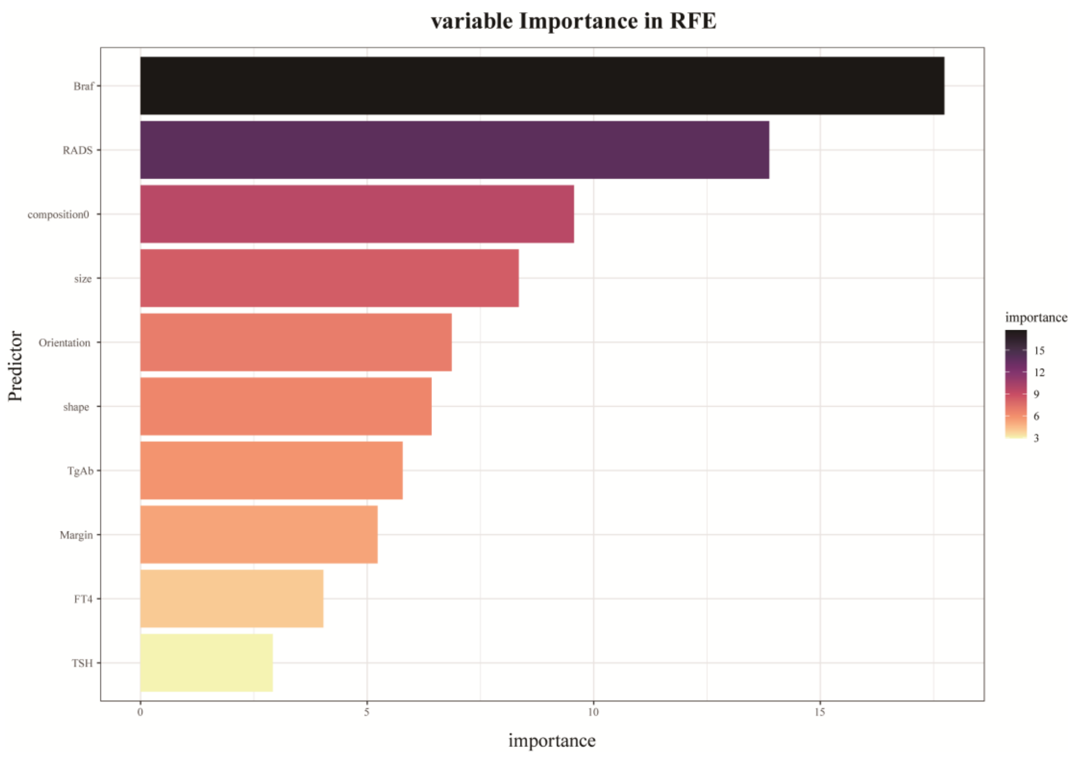

Fig. S5. Random Forest machine variable importance plot.

Abbreviations: TI-RADS, Thyroid Imaging Reporting and Data System; TSH, Thyroid-Stimulating Hormone; FT4, Serum-free Thyroxine; FT3, Serum-free Triiodothyronine; TgAb, Thyroglobulin Antibody; TPOAb, Thyroid Peroxidase Antibody.

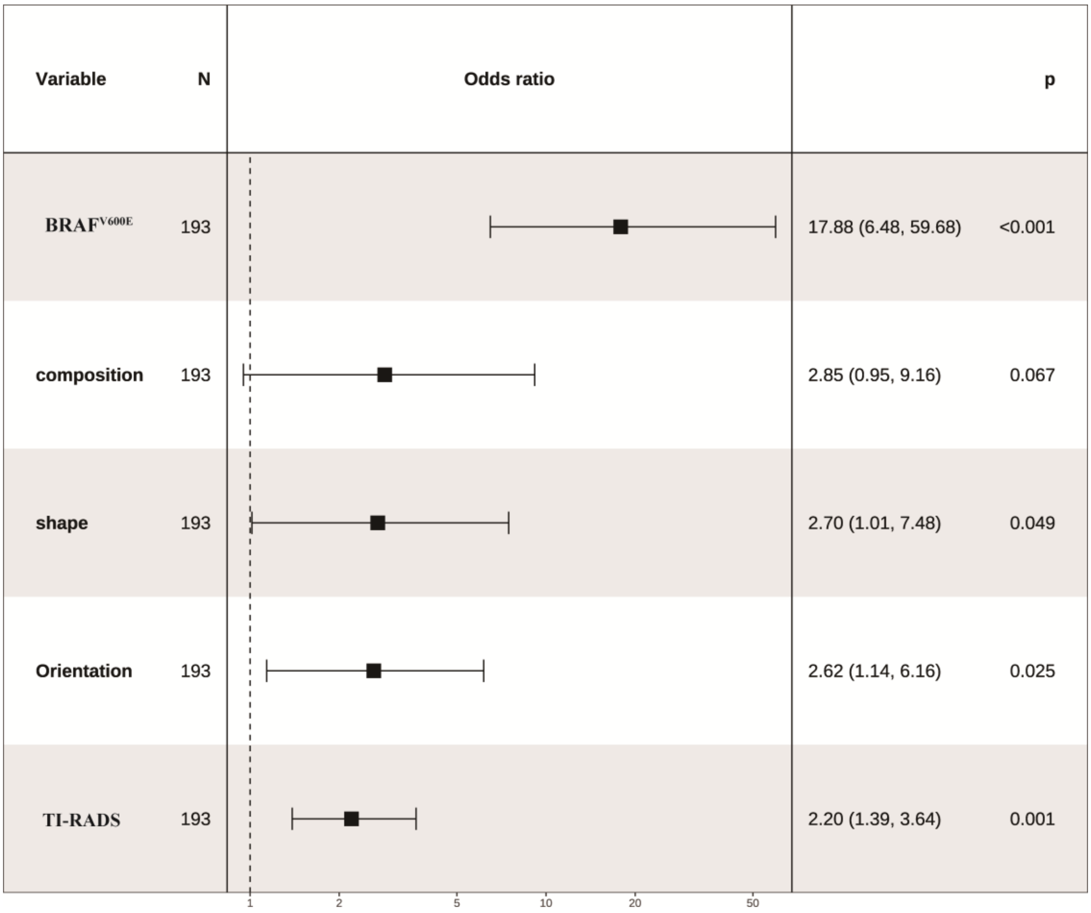

Fig. S6. Forest plot of the logistic regression model.

Abbreviations: TI-RADS, Thyroid Imaging Reporting and Data System.

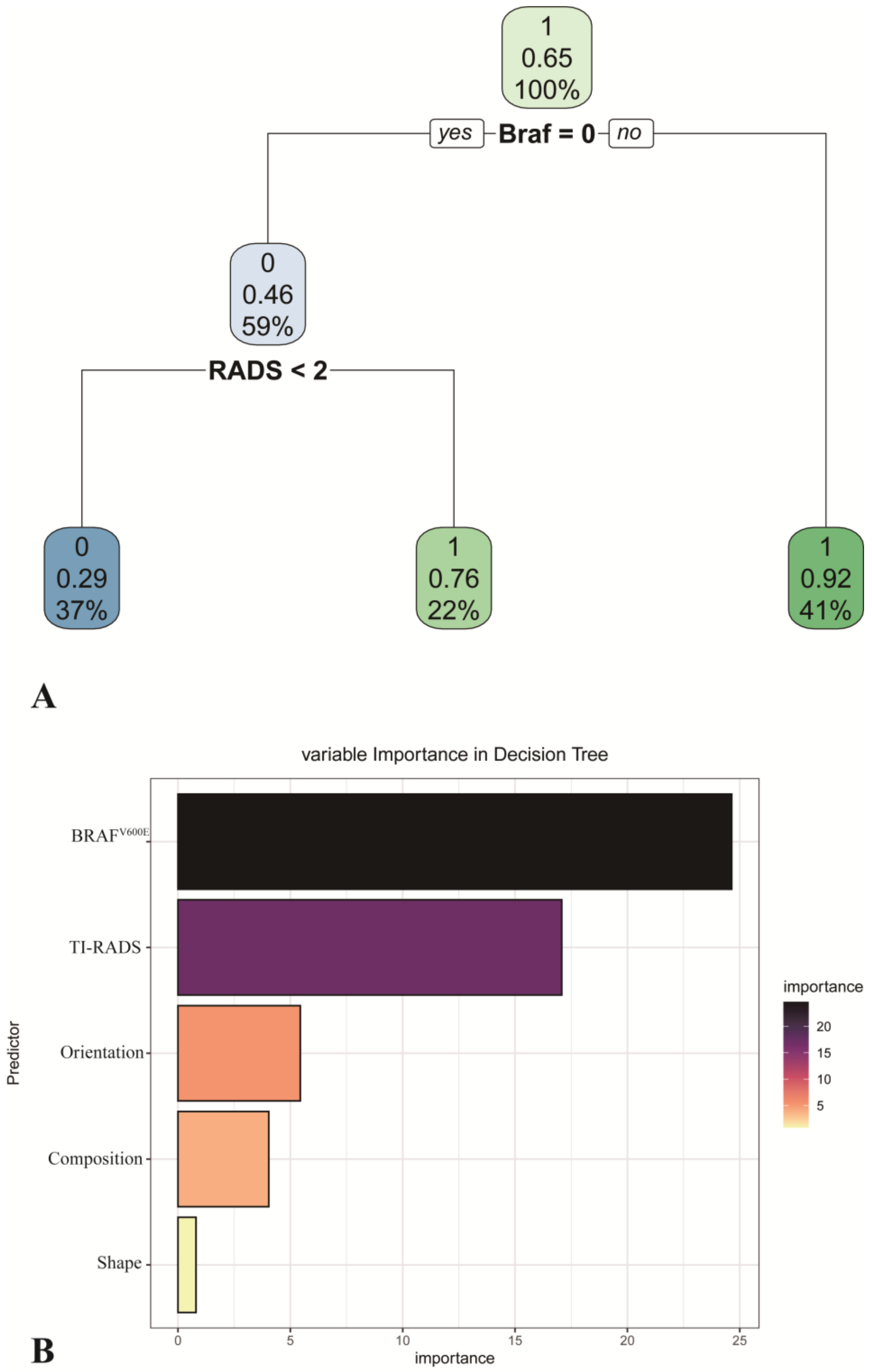

Fig. S7. Decision tree visualization and variable importance plots

Abbreviations: TI-RADS, Thyroid Imaging Reporting and Data System.

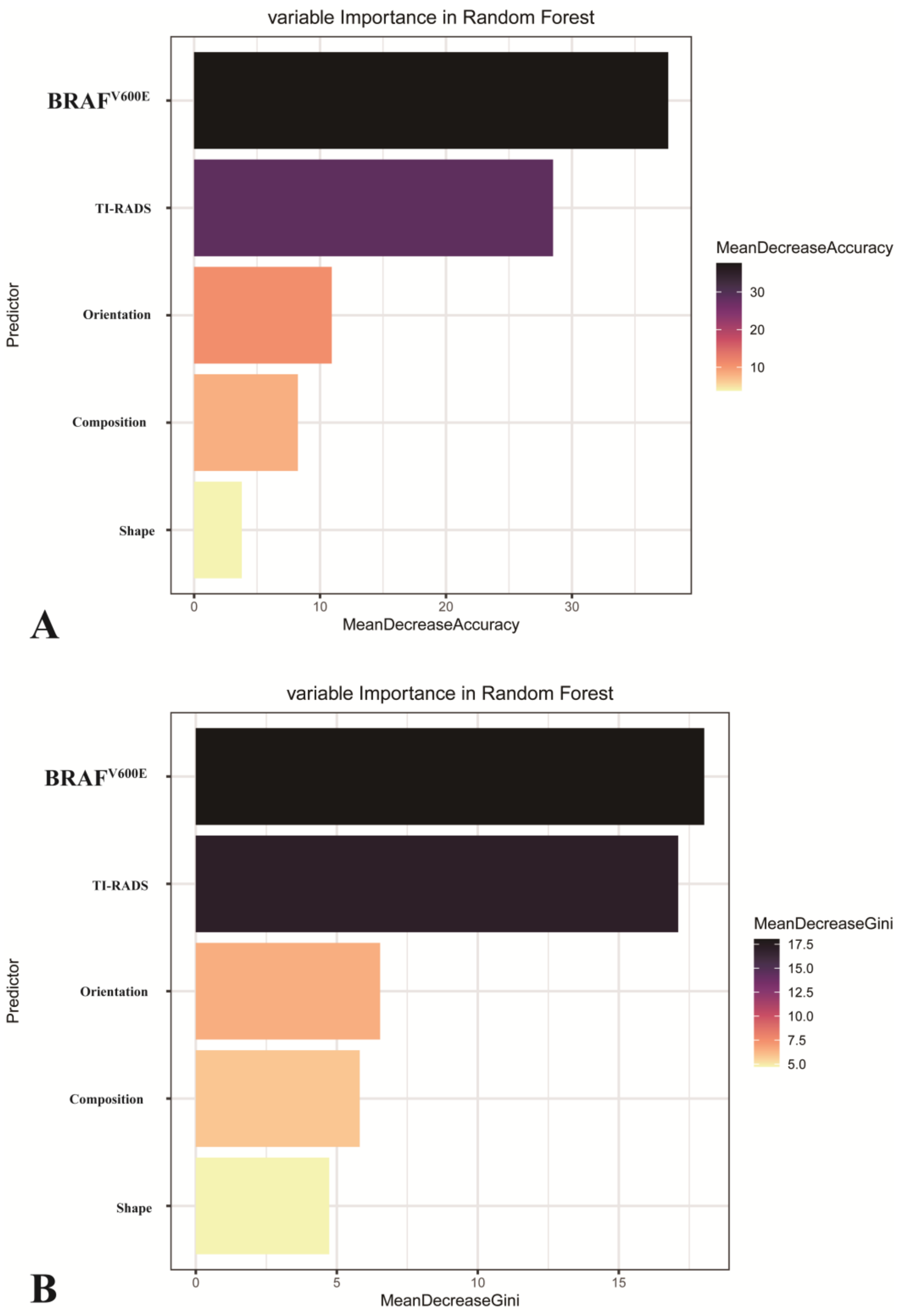

Fig. S8. Random forest variable importance plot

Abbreviations: TI-RADS, Thyroid Imaging Reporting and Data System.

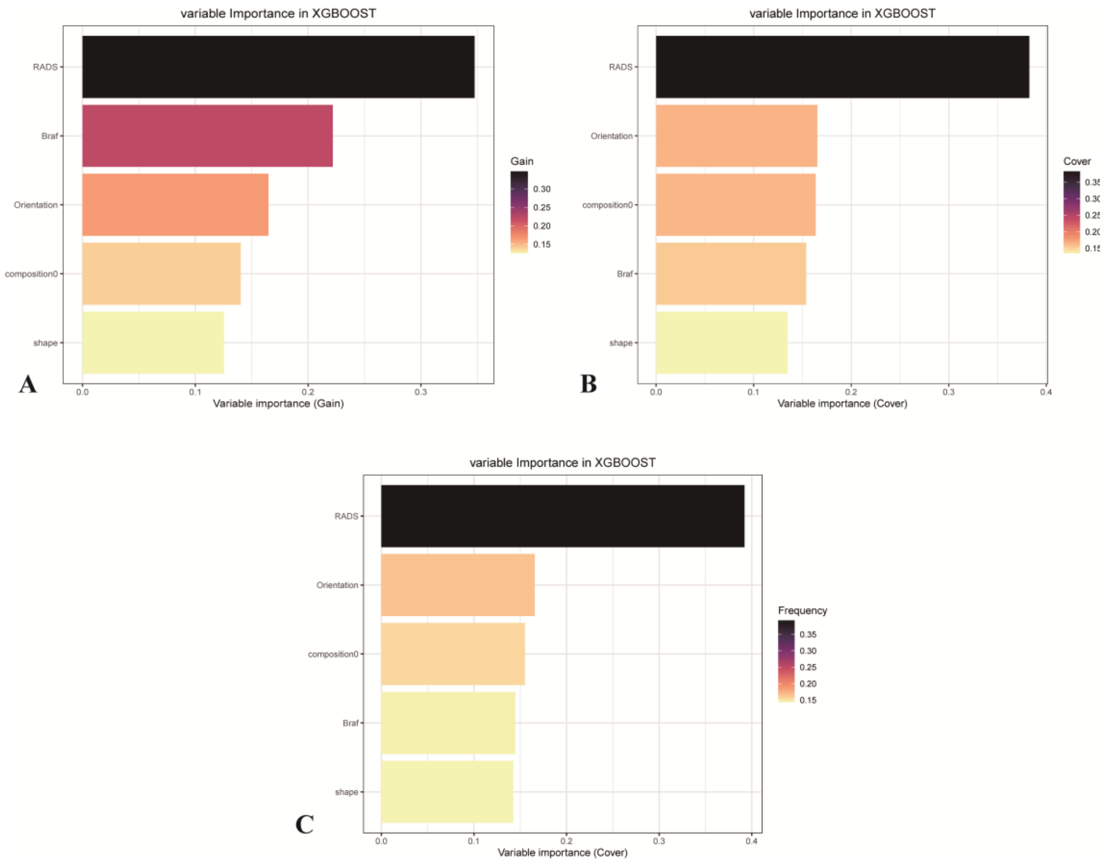

Fig. S9. XGBoost variable importance plot.

Abbreviations: TI-RADS, Thyroid Imaging Reporting and Data System.

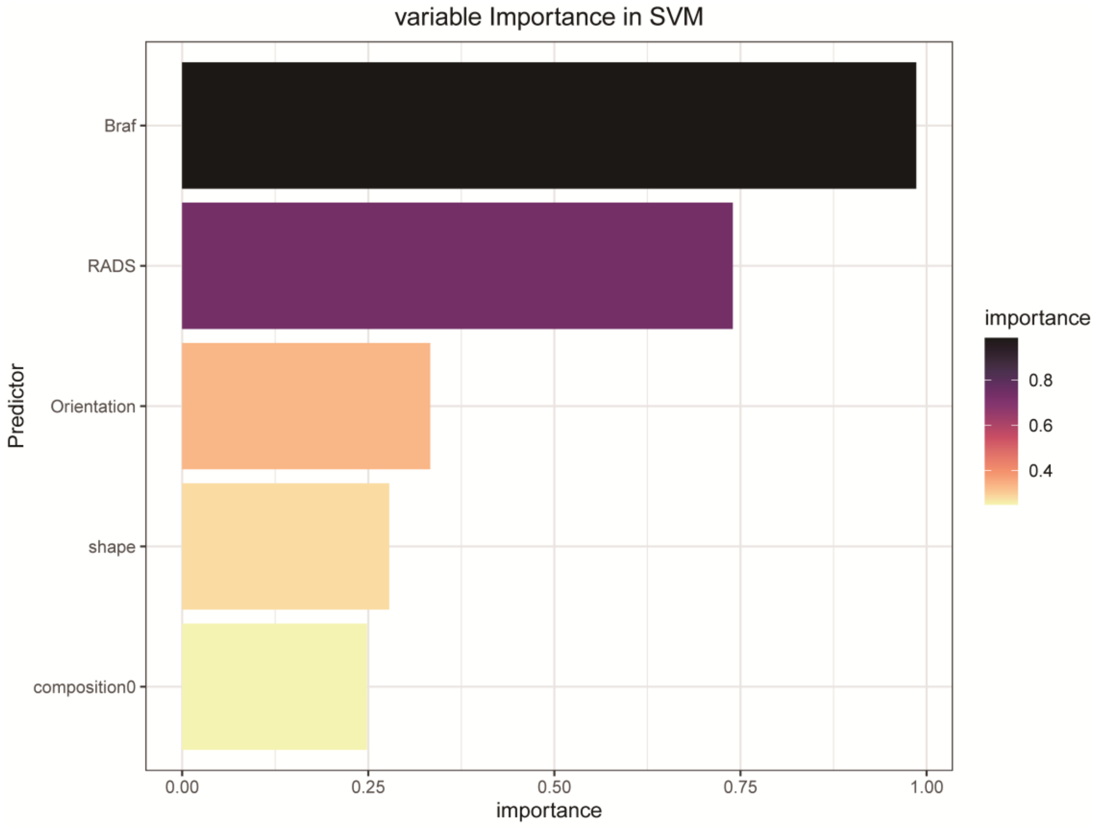

A

Fig. S10. Support vector machine variable importance plot.

Abbreviations: TI-RADS, Thyroid Imaging Reporting and Data System.

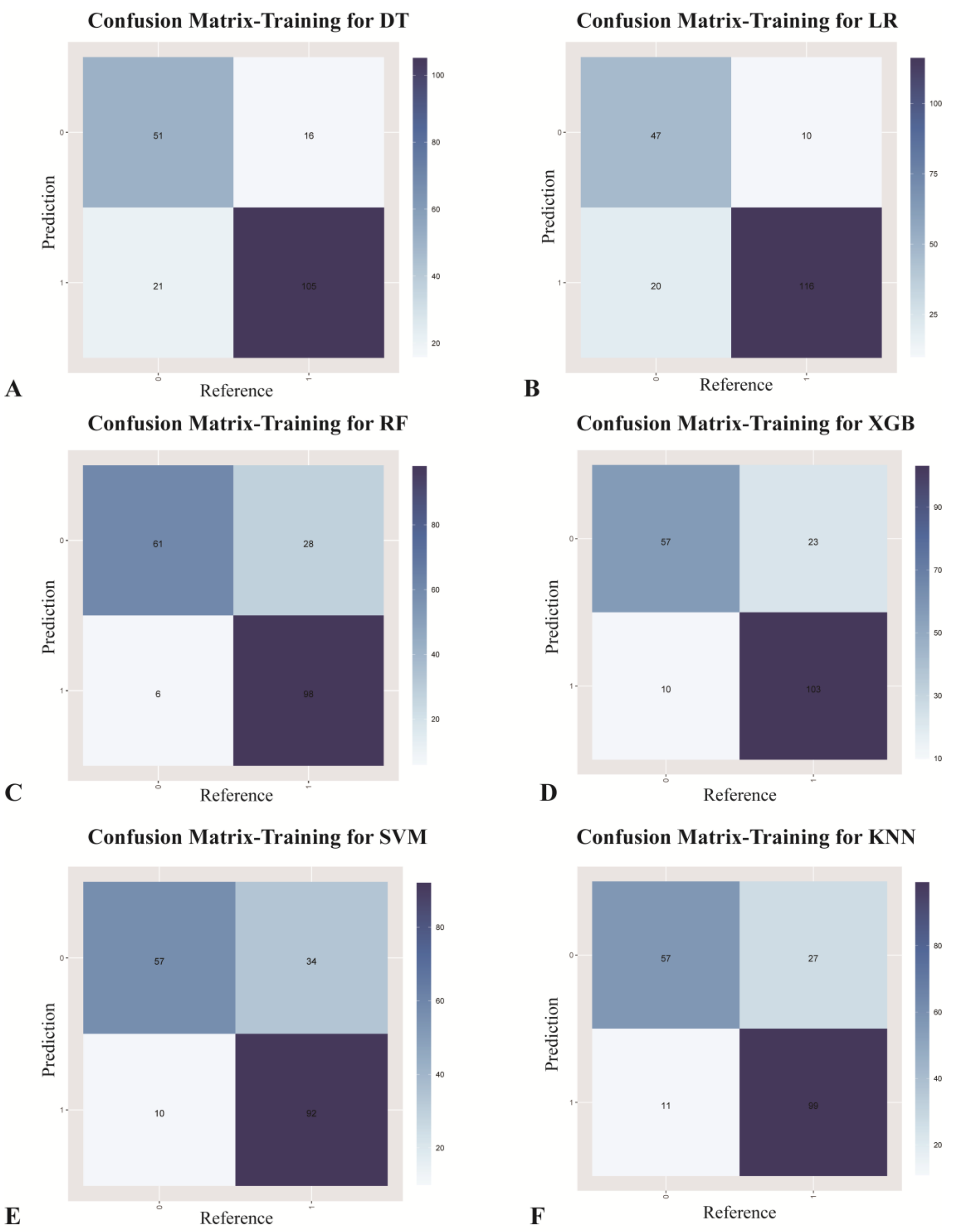

Fig. S11. The confusion matrix of the six machine learning models in the training set.

Abbreviations: LR, logistic regression; DT, decision tree; RF, random forest; XGBoost, extreme gradient boosting; SVM, support vector machine; KNN, k-nearest neighbours

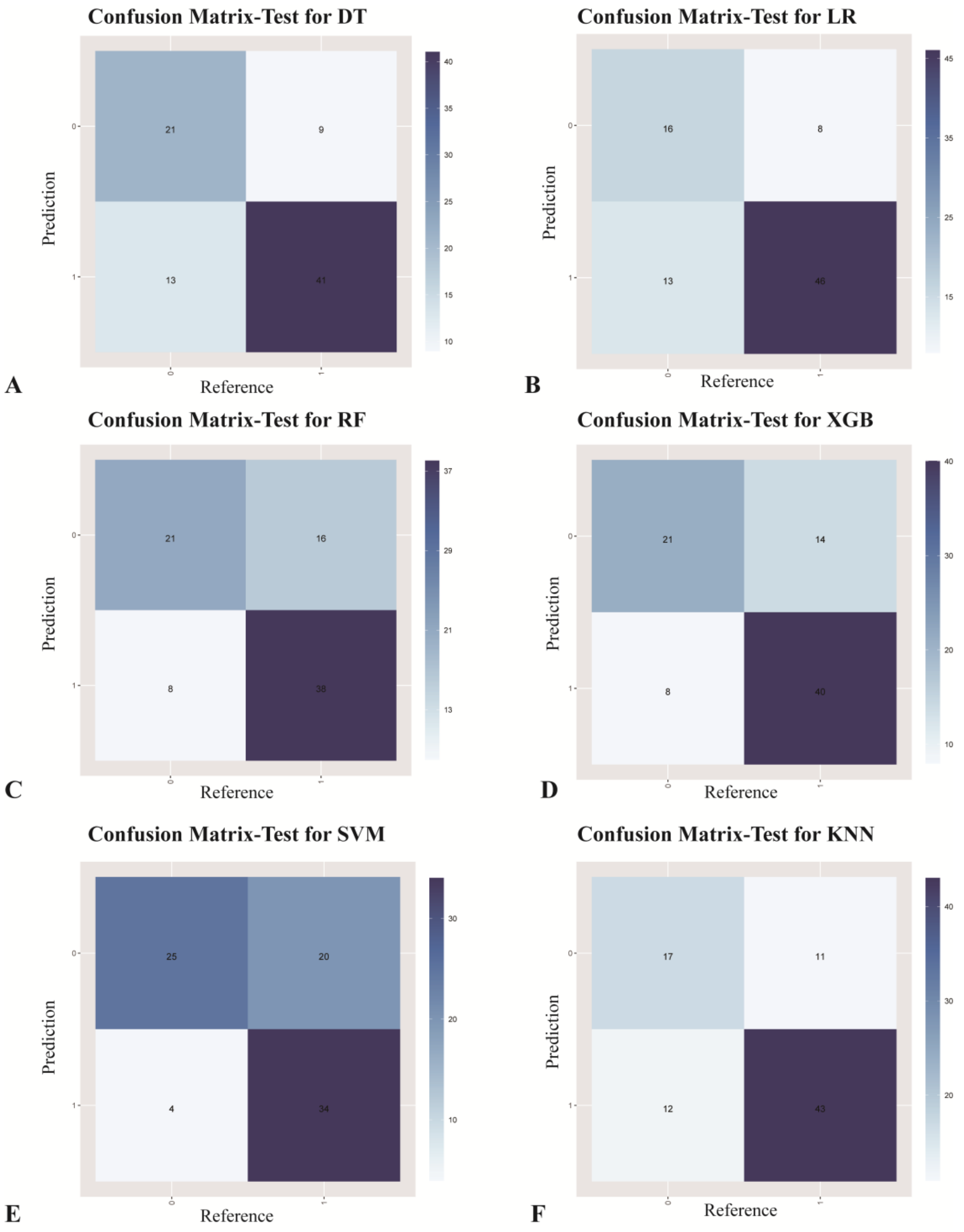

Fig.S12. The confusion matrix of the six machine learning models in the test set.

Abbreviations: LR, logistic regression; DT, decision tree; RF, random forest; XGBoost, extreme gradient boosting; SVM, support vector machine; KNN, k-nearest neighbours

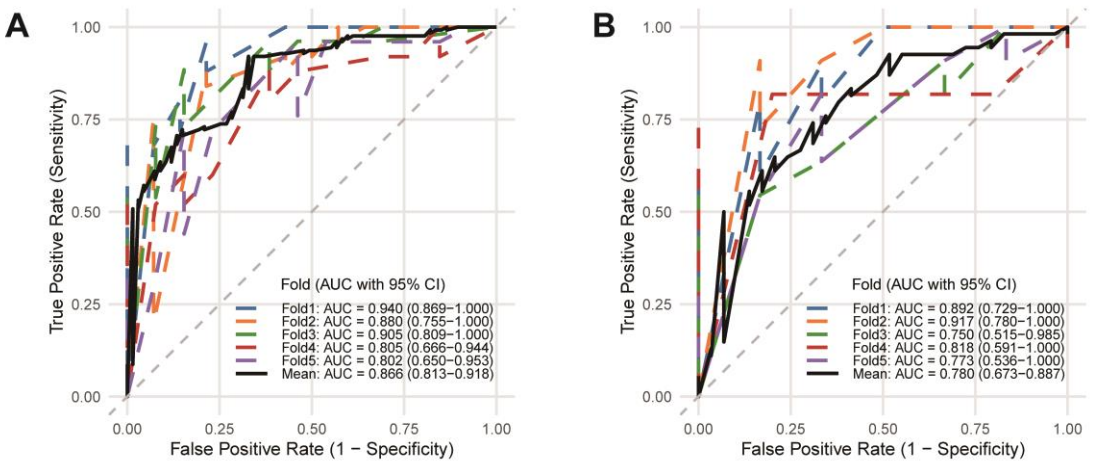

Fig.S13. 5-fold CV of LR models in the training set and validation set.

A ROC curves in the training set. **B** ROC curves in the validation set.

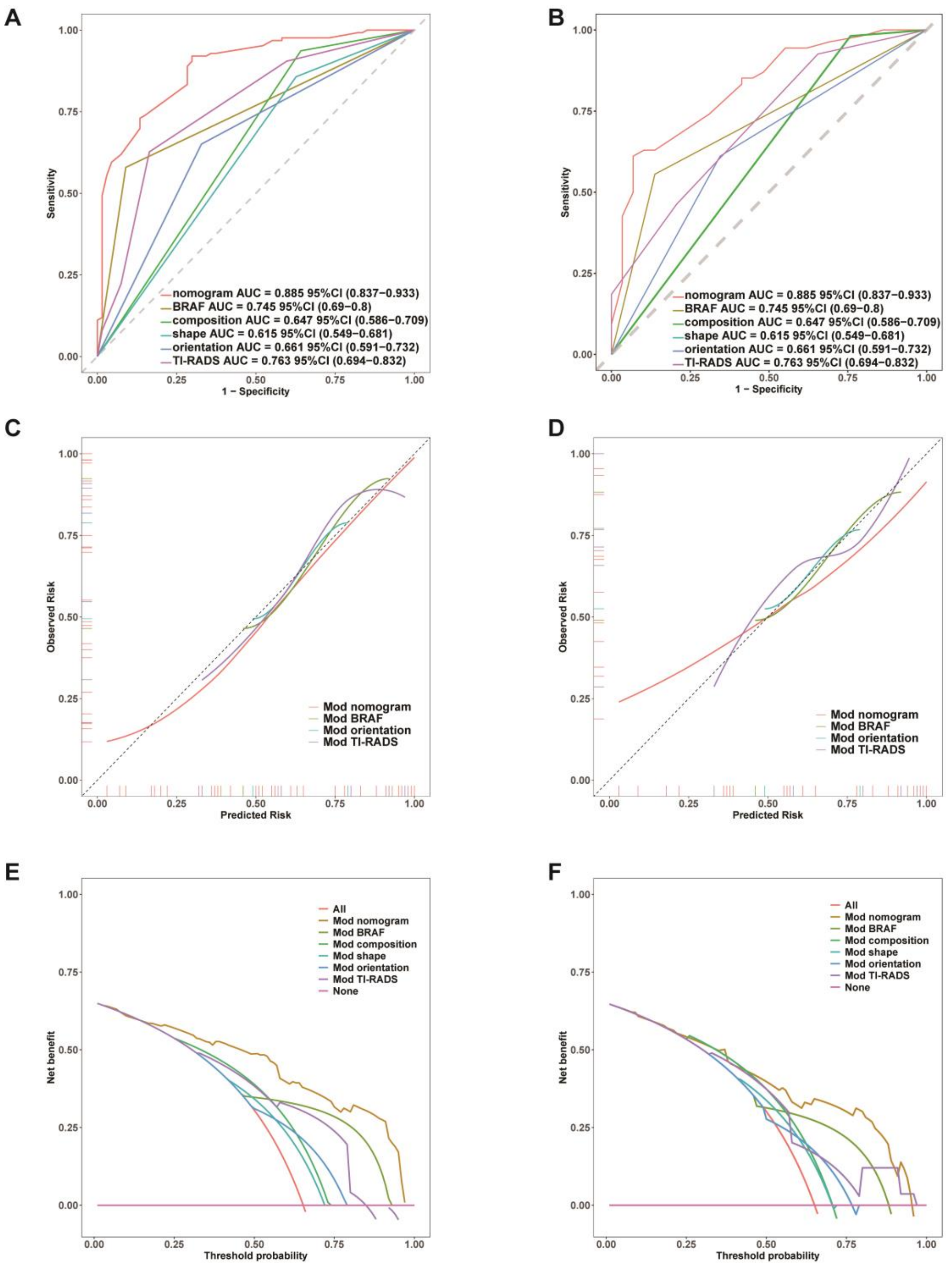

Fig.S14. Performance of the logistic regression model among nomogram, BRAF, composition, shape, orientation and TI-RADS.

A ROC curves in the training set. **B** ROC curves in the validation set. **C** Calibration curves in the training set. **D** Calibration curves in the validation set. **E** DCA results in the training set. **F** DCA results in the validation set.
